# Supplementary material for: Seeking diagnostic and prognostic biomarkers for childhood bacterial pneumonia in sub-Saharan Africa: study protocol for an observational study
Source: BMJ Open. 2021 Sep 30;11(9):e046590. doi: 10.1136/bmjopen-2020-046590 (PMC8487183; doi:10.1136/bmjopen-2020-046590)
Supplement: Author's manuscript [file bmjopen-2020-046590.draft_revisions.pdf]

BMJ Open

BMJ Open is committed to open peer review. As part of this commitment we make the peer review history of every article we publish publicly available.

When an article is published we post the peer reviewers' comments and the authors' responses online. We also post the versions of the paper that were used during peer review. These are the versions that the peer review comments apply to.

The versions of the paper that follow are the versions that were submitted during the peer review process. They are not the versions of record or the final published versions. They should not be cited or distributed as the published version of this manuscript.

BMJ Open is an open access journal and the full, final, typeset and author-corrected version of record of the manuscript is available on our site with no access controls, subscription charges or pay-per-view fees (http://bmjopen.bmj.com).

If you have any questions on BMJ Open's open peer review process please email info.bmjopen@bmj.com

A Study Protocol for an Observational Study Seeking Diagnostic and Prognostic Biomarkers for Childhood Bacterial Pneumonia in Sub-Saharan Africa

Journal:	*BMJ Open*
Manuscript ID	bmjopen-2020-046590
Article Type:	Protocol
Date Submitted by the Author:	03-Nov-2020

Complete List of Authors:	Valim, Clarissa; Boston University School of Public Health, Global Health<br>Olatunji, Yekin; Medical Research Council The Gambia<br>Isa, Yasir; Medical Research Council The Gambia<br>Salaudeen, Rasheed; Medical Research Council The Gambia<br>Golam, Sarwar; Medical Research Council The Gambia<br>Knol, Edward; University Medical Center Utrecht Department of Rheumatology and Clinical Immunology, Center of Translational Immunology<br>Kanyl, Sheriffo; Bansang Hospital<br>Jammeh, Abdoulle; Basse Hospital<br>Bassat, Quique; ISGlobal, Hospital Clínic - Universitat de Barcelona; Centro de Investigação em Saúde de Manhiça (CISM)<br>deJager, Wilco; University Medical Center Utrecht Department of Rheumatology and Clinical Immunology, Center of Translational Immunology; Luminex Corp<br>Diaz, Alejandro; Harvard Medical School, Department of Medicine; Brigham and Women's Hospital, Division of Pulmonary and Critical Care Medicine<br>Wiegand, Roger; Independent Researcher<br>Ramirez, Julio; University of Louisville, Division of Infectious Diseases<br>Moses, Marsha ; Children’s Hospital Boston, Vascular Biology Program; Harvard Medical School, Surgery<br>D'Alessandro, Umberto; Medical Research Council Unit, Disease Elimination and Control; London School of Hygiene and Tropical Medicine,<br>Hibberd, Patricia; Boston University School of Public Health<br>Mackenzie, Grant; Medical Research Council Unit The Gambia at the London School of Hygiene and Tropical Medicine; London School of Hygiene and Tropical Medicine Faculty of Infectious and Tropical Diseases, Disease Control

Keywords:	Respiratory infections < THORACIC MEDICINE, Diagnostic microbiology < INFECTIOUS DISEASES, Paediatric infectious disease & immunisation < PAEDIATRICS, Paediatric thoracic medicine < PAEDIATRICS

SCHOLARONE™
Manuscripts

I, the Submitting Author has the right to grant and does grant on behalf of all authors of the Work (as defined in the below author licence), an exclusive licence and/or a non-exclusive licence for contributions from authors who are: i) UK Crown employees; ii) where BMJ has agreed a CC-BY licence shall apply, and/or iii) in accordance with the terms applicable for US Federal Government officers or employees acting as part of their official duties; on a worldwide, perpetual, irrevocable, royalty-free basis to BMJ Publishing Group Ltd ("BMJ") its licensees and where the relevant Journal is co-owned by BMJ to the co-owners of the Journal, to publish the Work in this journal and any other BMJ products and to exploit all rights, as set out in our licence.

The Submitting Author accepts and understands that any supply made under these terms is made by BMJ to the Submitting Author unless you are acting as an employee on behalf of your employer or a postgraduate student of an affiliated institution which is paying any applicable article publishing charge ("APC") for Open Access articles. Where the Submitting Author wishes to make the Work available on an Open Access basis (and intends to pay the relevant APC), the terms of reuse of such Open Access shall be governed by a Creative Commons licence – details of these licences and which Creative Commons licence will apply to this Work are set out in our licence referred to above.

Other than as permitted in any relevant BMJ Author's Self Archiving Policies, I confirm this Work has not been accepted for publication elsewhere, is not being considered for publication elsewhere and does not duplicate material already published. I confirm all authors consent to publication of this Work and authorise the granting of this licence.

For peer review only

A Study Protocol for an Observational Study Seeking Diagnostic and Prognostic Biomarkers for Childhood Bacterial Pneumonia in Sub-Saharan Africa

Clarissa Valim[1], Yekin Ajauoi Olatunji[2], Yasir Shitu Isa[2], Rasheed Salaudeen[2], Sarwar Golam[2], Edward F. Knol[3], Sheriffo Kanyi[4], Abdoulie Jammeh[5], Quique Bassat[6-10], Wilco DeJager[3,11], Alejandro A. Diaz[12,13], Roger C. Wiegand[14], Julio A. Ramirez[15], Marsha A. Moses[16,17], Umberto D'Alessandro[2,18], Patricia Hibberd*[1], Grant Mackenzie*[2,18-20]

* These authors equally contributed to this work

AFFILIATIONS

1. Department of Global Health, Boston University School of Public Health, Boston, USA
2. Medical Research Council (MRC) Unit The Gambia at London School of Hygiene & Tropical Medicine, Fajara, The Gambia
3. Center of Translational Immunology, University Medical Center Utrecht, The Netherlands
4. Bansang Hospital, Central River Region, The Gambia
5. Basse Hospital, Upper River Region, The Gambia
6. ISGlobal, Hospital Clínic - Universitat de Barcelona, Barcelona, Spain
7. Centro de Investigação em Saúde de Manhiça (CISM), Maputo, Mozambique
8. ICREA, Pg. Lluís Companys 23, 08010 Barcelona, Spain.
9. Pediatric Infectious Diseases Unit, Pediatrics Department, Hospital Sant Joan de Déu (University of Barcelona), Barcelona, Spain
10. Consorcio de Investigación Biomédica en Red de Epidemiología y Salud Pública (CIBERESP), Madrid, Spain
11. Luminex corporation, Austin, Texas, USA
12. Department of Medicine, Harvard Medical School
13. Division of Pulmonary and Critical Care Medicine, Brigham and Women's Hospital, Harvard University, Boston, Massachusetts, USA
14. Independent Researcher, Boston, Massachusetts, USA

For peer review only

15. Division of Infectious Diseases, Department of Medicine, University of Louisville School of Medicine, Louisville, Kentucky
16. Vascular Biology Program, Children's Hospital Boston, Massachusetts, USA
17. Department of Surgery, Harvard Medical School, Massachusetts, USA
18. Department of Disease Control, Faculty of Infectious and Tropical Diseases, London School of Hygiene & Tropical Medicine, London, UK
19. Murdoch Children's Research Institute, Melbourne, Australia
20. Department of Paediatrics, University of Melbourne, Australia

Corresponding author:

Clarissa Valim

Dept. of Global Health, Boston University School of Public Health, Boston, Massachusetts, USA

cvalim@bu.edu

Keywords:

Biomarkers, pneumonia, bacterial infection, inflammatory proteins, machine learning

Word Count of the manuscript: 3,854

For peer review only

Abstract

Introduction:

Clinically diagnosed pneumonia in children is a leading cause of pediatric hospitalization and mortality. The etiology is usually bacterial or viral, but in endemic areas, malaria can cause a syndrome indistinguishable from clinical pneumonia. There is no accurate method to detect a bacterial infection in these patients and, as result, antibiotic therapy is frequently overprescribed, contributing to the spread of antibiotic resistance. Conversely, unrecognized concomitant bacterial infection in patients with malarial infections occur with omission of antibiotic therapy from patients with bacterial infections.

In Mozambique, we identified two combinations of blood proteins (signatures) with 96% sensitivity and 86% specificity for detecting bacterial disease. The current project aims to validate and improve these signatures by evaluating additional biomarkers in patients with clinical pneumonia. Our goal is to describe combinations of a limited number of proteins that would detect bacterial disease with high sensitivity and specificity. Furthermore, we seek to validate in a resource-limited setting signatures previously identified as accurate when tested in high-income countries, and to explore signatures to prognosticate clinical pneumonia.

Methods and analysis:

Patients (N = 900) aged 2-59 months presenting with clinical pneumonia at two Gambian hospitals will be enrolled and classified according to criteria for definitive bacterial etiology (based on microbiological tests and chest radiographs). We will measure proteins at admission using Luminex®-based immunoassays that include novel and known markers in 90 children with definitive and 160 with probable bacterial aetiology. We will also quantify proteins in 160 children classified according to the prognosis of their disease. Previously identified diagnostic

For peer review only

signatures will be assessed through accuracy measures. Moreover, we will seek new diagnostic and prognostic signatures through machine learning methods.

Results of this study may support development of a point-of-care test for bacterial disease to guide treatment and prioritization of intensive care in resource-limited areas.

Ethics and dissemination

Ethics approval for this study has been obtained from: the Gambia Government/MRCG Joint Ethics Committee (protocol 1616), and the Institutional Review Board of Boston University Medical Center (STUDY00000958). Study results will be disseminated to the staff of the study hospitals, in scientific seminars and meetings, and in per-reviewed publications.

Article Summary

Strengths and limitations of this study:

- ✓ This study aims to validate and improve upon previously identified signatures to detect bacterial infection and to explore prognostic signatures in a pediatric population with clinical pneumonia from a resource-limited country.
- ✓ To improve upon diagnostic and seek prognostic signatures, we will quantify known and recently identified biomarkers, and we will use different machine learning approaches penalized when missing a bacterial infection.
- ✓ Results of this study should allow future development of a point-of-care (POC) test using existing technologies that can result in substantial improvement in care of patients with clinical pneumonia by reducing over/under use of antibiotic therapy, particularly in resource-limited areas.
- ✓ A difficulty shared by studies aiming to identify biomarkers to diagnose bacterial infection is lack of an optimal gold standard for bacterial infection, thus, as others working in this

For peer review only

field have done, we will use a composite criteria based on microbiological and radiological evidence of infection (primary criteria) and probable criteria based on expert evaluation of clinical and laboratory data.

- ✓ Although the sample size is relatively small, results will establish a benchmark against which the identified signatures in populations in other areas and in different subgroups, such as children with undernutrition, can be compared.

For peer review only

Introduction

Clinical pneumonia may be caused by viral or bacterial infections and, where malaria is endemic, malaria can cause a syndrome indistinguishable from clinical pneumonia[1]. There has been a growing number of publications on biomarkers for the diagnosis of bacterial infections and to prognosticate outcomes in patients with clinical pneumonia[2-36]. Such increased attention is driven by the need for low cost point-of-care (POC) tests able to accurately, reliably, and promptly diagnose bacterial infections that can guide treatment [37-40]. All existing tests have delayed turn-around times or require specialized facilities that are often unavailable in resource-limited settings. POC tests can support timely decisions about treatment and ameliorate the overprescription of antibiotics to treat viral infections, thus, slowing the progression of global antibiotic resistance[41-45]. In malaria endemic areas, antibiotics may also be “underprescribed” and children with bacterial pneumonia may be mistakenly treated for malaria and not receive antibiotic therapy[42 46]. Prognostic POCs could also guide referrals and allow prioritization of intensive treatment measures that are particularly scarce in resource-limited settings.

Symptoms of bacterial and viral diseases and of malaria often overlap[47 48]. Radiographic evidence of pneumonia has been used as an endpoint in studies of pneumococcal vaccine, but chest X-rays, particularly in resource-limited settings, when available have moderate reliability [49-57], and may result in both false positive and negative results. Laboratory tests have low accuracy. Blood or pleural fluid cultures are highly specific for diagnosing bacterial infections but have low sensitivity and results are not promptly available[58 59]. Bacterial and viral polymerase chain reaction (PCR) tests require specialized resources, and their accuracy is often too low to reliably and accurately guide treatment[48 60-62]. Furthermore, in children, antigen detection in the urine has limited utility and induced sputum samples are difficult to obtain and likely to be contaminated with the normal flora[63-66]. Where malaria is endemic, standard malaria diagnostic

For peer review only

tests can detect the parasite in blood. Nevertheless, malaria may co-exist with other infections while not causing the respiratory syndrome[67 68].

Generally, previous biomarker studies to diagnose bacterial diseases or to prognosticate disease in patients with pneumonia or sepsis focused on procalcitonin (PCT) and/or C-reactive protein (CRP) and concluded that the markers had moderate accuracy (area under the receiver operating characteristic curve [AUC-ROC]< 0.80)[8-13 33-35 69-92]. Accuracy of a single biomarker in bacterial pneumonia is probably low, since many biomarkers may be in the inflammatory pathway triggered by several pathogens[93]. More recently, studies screened through novel biomarkers with promising results[94 95]. Particularly, a biomarker signature based on haptoglobin (HP) and lipocalin (NGAL) has been identified to differentiate malaria and probable bacteria etiology in pediatric patients with clinical pneumonia living in a resource-limited country[96]. Two other promising signatures to differentiate viral from bacterial infections in high-income countries (HIC) have been identified. The first (ImmunoXpert) is based on CRP, Tumor Necrosis Factor-related apoptosis-inducing ligand (TRAIL) and Interferon Gamma-Induced Protein 10 (IP-10) and has been validated in HICs, with sensitivity ranging from 87% to 94% and specificity from 90% to 94%[4 97-100].The second is based on CRP and Mixovirus Resistance Protein A (MxA) and has been implemented in a POC (FebriDx) in HICs, with sensitivity and specificity varying from 80% to 96% and 67% to 94%, respectively. [3 101 102]. Although promising, these two signatures need to be validated in resource-limited settings where inflammatory responses may vary because of malaria, co-infections, and undernutrition[103].

In Mozambique, we scanned through several biomarkers and combined them through machine learning methods aiming to detect bacterial infection (versus no bacterial infection) in pediatric patients with clinical pneumonia. Considering the potential of co-existing malaria infections, we sought biomarker signatures by comparing children with malaria, bacterial and viral infections

For peer review only

and validated accurate signatures by comparing patients with and without bacterial infections[104]. Signatures with 3-5 proteins were identified that had high sensitivity for the diagnosis of bacterial pneumonia (96-91%), while misdiagnosing few viral cases. The selected signatures performed better than signatures including commonly collected clinical and laboratory markers, as well as pneumonia with consolidation on X-ray. The current study builds on our previous findings and aims to validate previous diagnostic signatures identified by us and other groups, and in addition to improve upon signatures by scanning through novel biomarkers in a different population in rural Gambia.

Biomarkers for signatures will be selected and combined using a comprehensive set of data mining approaches. To validate and improve upon prior signatures and seek prognostic signatures we will add some novel promising biomarkers such as resistin and lactoferrin[36 105 106]. At the end of this study, we expect to select signatures with excellent accuracy (≥ 93% sensitivity), that have the highest prognostic utility, and be comprised of a limited number of biomarkers that can be developed in a POC test. Because of the lack of accurate diagnostic tests, there is no adequate gold standard for biomarker research. To tackle these difficulties, we will use two different diagnostic standards: "definitive" based on pathogen detection and "probable" based on clinical and radiographic findings[97 107 108]. Our focus is on identifying patients who need antibiotic therapy for bacterial diseases.

Study objectives

Specifically, this study aims to:

I. Assess combinations of blood proteins to accurately and reliably diagnose bacterial pneumonia in pediatric patients.

We expect that protein combinations that were identified in previous studies as accurate for diagnosing bacterial infection in patients with clinical pneumonia will have high sensitivity,

For peer review only

specificity, and AUC-ROC in an African pediatric population different from the Mozambican children. Biomarkers of these signatures will be absent in healthy children. Moreover, we hypothesize that accuracy of previous signatures can be improved by seeking to combine them with novel candidate proteins.

II. *Explore prognostic biomarker signatures in patients with clinical pneumonia caused by any pathogen.*

We anticipate that combination of inflammatory proteins in the blood measured at admission will accurately identify patients who will clinically deteriorate.

Methods and Analysis

Design

To test existing and identify novel diagnostic biomarker signatures for bacterial disease, we will measure proteins in the blood at admission and collect clinical and diagnostic laboratory information at admission to ascertain diagnostic groups. Diagnostic signatures will be sought by comparing children in the target diagnosis groups. To seek prognostic biomarker signatures, we will prospectively follow clinical outcomes collected after admission and discharge. When studying prognostic signatures in the primary analysis, proteins will be compared across three groups with poor, median and good prognosis.

Study Environment

The study will be conducted at the Boston University School of Public Health (BUSPH), the Medical Research Council Unit The Gambia at London School of Hygiene & Tropical Medicine (MRCG), and the Multiplex Core Facility of the Center of Translational Immunology from the University Medical Center Utrecht (CTI/UMC). Patients will be recruited at the Basse and Bansang Hospitals. These hospitals serve a population of 178,510 (224 villages) and 99,113

For peer review only

(217 villages), respectively, with 19% of the population aged <5 years. Diagnostic laboratory tests will be conducted at the MRCG, Basse Field Station. The multiplex bead-based immunoassay will be conducted at CTI/UMC and analysis will be conducted at BUSPH.

Outcomes

Two sets of outcomes will be evaluated, one for each study aim:

Objective I - Diagnostic Group Classification

The primary objective of the diagnostic group classification is to identify patients in need of antibiotic therapy for a bacterial infection and not to identify the pathogen responsible for the respiratory symptoms. Patients will be assigned to bacterial, malarial and viral groups according to a definitive classification (primary analysis) (Fig. 1) when seeking signatures, and bacterial and non-bacterial infection groups when testing signatures. Patients will also be categorized into bacterial and non-bacterial infection groups based on a probable classification criterion (secondary analysis). Patients with ambiguous diagnosis who do not meet our criteria will be excluded from the study of Objective 1.

For the definitive classification, a patient will be assigned to the bacterial (BA) group if he/she has a positive bacterial pathogen culture of fluid from a normally sterile site (e.g. blood or pleural fluid). Patients will be assigned to the viral (VI) group if they have negative bacteria microbiological tests, negative malaria blood slides, X-ray without "endpoint pneumonia" (consolidation or pleural effusion[50]), no evidence of fungal infection, and positive PCR for a viral pathogen from nasopharyngeal swabs (NPS). Patients will be assigned to the malarial (MA) group if they have normal X-ray (with neither infiltrates nor endpoint pneumonia), no bacterial infection and > 0/µl asexual *P. falciparum* parasites if they are aged < 1 year, or > 2,500 asexual parasites/µl of blood if they are aged > 1 year. Patients who are admitted with viral infections

For peer review only

but who develop a secondary bacterial pneumonia during hospitalization will be excluded from the VI group.

Patients with HIV infection or exposure (< 1 year old) will be categorized as fungal pneumonia if they have chest X-ray with bilateral/symmetrical reticular or granular opacities without lobar consolidation and severe hypoxia (≤ 85% at admission) or positive NPS PCR for *Pneumocystis jiroveci* or a blood culture positive to any pathogenic fungal microorganism.

For the diagnostic categorization of probable bacterial pneumonia, patients will be assigned to the probable BA group based on review of data by an expert panel including pediatricians and infectious diseases specialists with at least 10 years of experience. Data will include clinical information collected at and during admission, Chest X-ray results, full blood cell counts, malaria microscopy and/or rapid diagnostic test (mRDT), bacteria culture, viral NPS, and other laboratory test results, when available, e.g., urinalysis. Two experts will review patient data, independently and blindly, classifying patients as “certainly” BA or non-bacteria (NBA), “probable” BA or NBA, “unclear” BA or NBA. Only patients with consensual “certainly” or “probable” diagnosis will be included in this analysis.

Objective II - Poor Prognosis

Poor prognosis will be primarily based on a three-category outcome recorded during and after hospitalization. Additionally, we will explore secondary outcomes associated with a poor evolution of the clinical pneumonia during the first 5 days of hospitalization, at discharge and 30 days after admission.

For the three-category outcome, regardless of the original infection, patients will be selected from the following three groups: a) children who die during or up to 30 days after admission; b)

For peer review only

children with prolonged hospital stay (> 7 days) or who need to have antibiotic therapy changed within 48 hours of admission, or who are re-admitted within 30 days after the first admission with symptoms associated with the first admission; and c) children discharged well within 3 days of admission and without the need for a change in antibiotic therapy after admission. Secondary outcomes of clinical deterioration will be: a) area under the first five days of the oxygen saturation curve; b) necessity to change antibiotic therapy within the first 2 days from admission or to receive oxygen therapy when it was not required at admission; c) duration of hospital admission.

Participants

We will enroll 900 patients with clinical pneumonia aged 2-59 months by January of 2021 assuming we will have approximately 30 patients with a definitive diagnosis of bacterial infection, 30 with malaria, and 30 with viral infections (Fig. 1). Among the 900 patients, we anticipate enrolling 60 and 100 patients with and without probable bacterial infection, respectively.

For the study of prognostic biomarkers (Fig. 2), we will include 160 patients. All deaths during or up to 30 days from admission will be selected and the remaining sample size will be divided between the other two category outcomes, i.e., (b) prolonged admission, readmission or changed antibiotics during admission, and (c) discharged within 3 days with improvement of symptoms. To meet the target sample size in the groups (b) and (c), eligible children will be randomly selected.

A total of 20 community healthy control children aged 2-59 months without any symptoms or signs and no malaria infection will be enrolled in vaccination clinics in Basse to assess precision of signatures.

For peer review only - http://bmjopen.bmj.com/site/about/guidelines.xhtml

Patient and public were not involved in the design of this research.

Inclusion and Exclusion criteria

Pediatric patients aged 2-59 months will be screened and will be enrolled if they present with cough or difficult breathing, the guardians provide informed consent, are referred for probable admission, and have at least one of the following signs: increased respiratory rate for age based on the World Health Organization (WHO) criteria, lower chest indrawing, peripheral arterial oxygen saturation $< 93\%$ (measured through pulse oximetry), grunting, nasal flaring, or undernutrition. The WHO criteria for increased respiratory rate are: respiratory rate ≥ 50 for child 2-11 month old; and respiratory rate ≥ 40 for child 1-5 years old. Undernutrition will be defined as: visible wasting OR middle upper arm circumference (MUAC) < 11.5 cm if aged ≥ 6 months OR weight-for-height Z-scores < -3. Patients will be excluded if they have suspected tuberculosis based on a cough lasting > 2 weeks, were admitted to a hospital in the previous two weeks, and have evidence of any condition that could be worsened by collection of blood.

Healthy community controls will be enrolled if: a mRDT is negative, vaccines have not been administered that day, there are no symptoms of any disease, and no history of hospital admission in the previous 4 weeks. Healthy controls will be excluded if they are later found to have malaria infection detected by a positive microscopy.

Recruitment and follow-up

Upon arrival at any of the study hospitals, patients aged 2-59 months will be screened for the presence of respiratory symptoms and undernutrition by a Research Nurse (Fig 2). If they pass this first level assessment, they will be referred to a second Research Nurse for a detailed

For peer review only

medical history and physical examination. Those patients that are considered eligible will undergo informed consent procedures.

After informed consent, patients will be referred for a thorough clinical examination and admission procedures by a clinician and then for sample collection and chest X-ray. NPS will be performed and 5 mL of venous blood will be drawn (never more than 3 mL/kg per WHO standard[109]) with 3 mL immediately placed into one pediatric blood culture bottle (Pedibact®; BD, Franklin Lakes, NJ, USA), and the remaining 2 mL placed in an EDTA vacutainer. From the EDTA tube, a drop of blood will be used for hemoglobin rapid test and mRDT, both part of standard of care in the Gambia. Other tests based on standard of care may be conducted, including urinalyses, glucose, examination of CSF and pleural fluid, or lung aspirate. HIV testing and treatment. Blood in EDTA will be processed and used within one hour from collection for full blood cell count, malaria microscopy, and will be centrifuged and aliquoted to store plasma at -80C in the MRCG biobank for the multiplex bead-based immunoassay.

Follow-up

After admission, patients will be followed daily for 5 days to record: pulse oximetry, antibiotic therapy, feeding, clinical deterioration with need for additional tests. If patients are receiving oxygen during the follow-up visits, they will have their lowest recorded pulse oximetry imputed. At hospital separation, a Research Nurse will fill a CRF to record diagnosis and outcome (e.g. death, absconding or discharge alive and well). Moreover, for patients who are discharged, a phone call will be placed to the parent/guardian 30 days after admission.

Study measurements and procedures

- <u>Bacterial Culture:</u> Approximately 3 mL will be incubated in an automatic BACTEC® 9050 system (BD) for a minimum of 4 days. Positive blood cultures will be examined

For peer review only

by Gram stain and sub-cultured onto blood agar, chocolate agar or MacConkey agar plates based on Gram stain. Bacterial growth will be defined as contaminant when bacteria generally considered skin flora are isolated from the positive blood culture (e.g., Coagulase–negative Staphylococci, Bacillus species or Micrococci) [110]. Lung fluids will be cultured according to procedures previously described[111].

- Culture of CSF, lung aspirate and pleural fluid aspirate: Cell count and direct gram reaction will be performed as well as bacterial antigen latex agglutination test. A 50 to 100µl aliquot of the specimen will be inoculated onto Blood Agar, Chocolate Agar and MacConkey Agar plates for isolation of bacterial growth.

.

- Viral PCR of NPS: NPS will be placed in media that will be aliquoted and stored at -80˚C for rtPCR. Assays will use primers and probes for influenza viruses types A and B, RSV types A and B, adenovirus, parainfluenza viruses 1, 2, 3 and 4, human metapneumovirus, coronavirus, bocavirus, swine flu virus (H1N1) and rhinovirus. *P. jiroveci* will also be screened in this PCR. Viral RNA and DNA will be extracted from supernatant of NPS samples using the QIAamp® cador® Pathogen Mini Kit or the Qiacube® HT manufactured by Qiagen and PCR reactions will be ran as described elsewhere[112].

- Malarial Microscopy: Thick blood smear slides will be read blindly by two microscopists and, if discrepant, by a third.

- Full blood cell count (FBC): FBC will be performed on a Medonic M51 5-part hematology analyzer (Boule Diagnostics AB, Domnarvsgatan, Spanga, Sweden).

For peer review only

- Multiplex Bead-Based Immunoassay: Using Luminex® technology will quantify proteins in plasma and will be conducted at the Multiplex Core Facility of CTI/UMC ISO9001:2015 laboratory using FlexMAP3D systems[113]. A total of 50 proteins will be included in the assay: a) proteins identified in our previous study to constitute accurate signatures (e.g., Haptoglobin, IL-10 and TIMP-1, and Resistin)[114]; b) proteins that discriminated the three groups in high-throughput scans conducted previously (e.g., BPI, EPHB2, LTF); c) proteins specified in previous studies and listed above (e.g., lipocalin, PCT, TRAIL, and IP-10).

- Chest Radiographs: Frontal and lateral radiographies will be obtained, and images will be recorded in a computer file. Radiographic findings will be classified as proposed by Cherian et al[50] into presence of consolidation, pleural fluid, other infiltrates, or normal. Additionally, the presence of reticular/reticulonodular patterns, cavities, pneumatoceles, lymphadenopathy, abscess, focal fibrosis, pneumothorax, cardiomegaly, and foreign body will be noted. Chest X-rays will be read by two clinicians and, if discrepant, they will be adjudicated by a third reader, all with training in radiographical interpretation for pneumonia and readings calibrated before the study starts. Also a pulmonologist, will read a sample of 10% of X-rays for quality assurance and control.

Data management

All data will be captured through laptops/desktop computers using an Electronic Medical Record System (EMRS) developed at the MRCG. The only exception will be for follow-up of admitted patients that will be captured on paper forms and later entered in the EMRS. During power outages, paper forms will be completed and when electricity is back, data will be entered into the EMRS. To ensure data quality, data validation rules have been established in the EMRS

For peer review only

that generates alert messages. Additionally, a complete process flow has been established to sequentially activate forms for entry and a report listing missing variables is generated during entry of forms to assure form completeness.

Data analysis

Descriptive analysis will be conducted to seek outliers, assess assumptions for tests, and appropriate transformations. Patient severity scores at baseline will be derived based on principal component analysis (PCA), including baseline clinical and laboratory variables. These scores will be included in the analysis and used to explore stratification of patients in the study of diagnostic biomarker signatures. Adjustments for potential confounders (e.g. overall and cell-specific leukocyte counts; breast feeding; undernutrition) and assessment of heterogeneity across subgroups will be done by incorporating the factor in analysis.

In Analysis of Objective 1, we will first test previous diagnostic biomarker signatures[4 104] through estimating AUC-ROC, sensitivity, and specificity with 95% CI. Using levels of biomarkers included in Valim et al[104] and Oved et al[97] we will reproduce signatures using the original models. To discover alternative biomarker signatures, we will start by conducting single biomarker analysis in which we will rank individual markers by pairwise comparisons of the three definitive diagnosis groups (primary analysis) and the two bacterial vs. non-bacterial probable diagnosis groups (secondary analysis) and estimation of AUC-ROC with 95% confidence interval (CI)[115]. We will also estimate sensitivity and specificity for an optimal cutoff (weighting towards high sensitivity to bacteria) based on comparison of the BA and NBA definitive diagnosis groups through resampling. Next, to identify composite biomarkers, all markers (including clinical and laboratory tests and severity scores) will be combined and selected through comparison of the three definitive diagnosis groups using: logistic regression with elastic net; classification or regression trees depending on the specific outcome; and

For peer review only - http://bmjopen.bmj.com/site/about/guidelines.xhtml

support vector machine (SVM). In SVM, non-linear radial basis function kernels will be used and features will be selected through a penalization and recursive feature elimination[116 117]. We will train models and identify signatures based on the three diagnosis groups although the focus is in classification of BA and NBA (defined as MA or VI diagnosis) because using three groups, signatures are likely to be more accurate when markers are not monotonically increasing or decreasing across groups. For instance, if a marker is high in BA, low in VI and high in MA, levels of VI with MA are sub-optimally averaged when VI and MA are collapsed for training signatures. Within each class of models, tuning parameters will be obtained through a metric of misclassification (e.g., deviance or error) estimated in 5-fold cross validation. Additionally, models will be selected based on a biased choice of tuning parameters (those determining ≤ 5 markers). Penalties for missing a bacterial infection will be used. Models will be tested by estimating accuracy to diagnose BA vs. non-BA in the definitive diagnosis classification using resampling. Moreover, accuracy will be estimated by comparing BA with NBA probable diagnosis groups.

Analyses of Objective 2 will use categorical or continuous surrogates of poor prognosis as outcomes and markers as predictors. Markers will be measured at admission. Signatures will be sought and tested using the same models of Objective 1.

To estimate precision, we will report the coefficient of variation (CV) of selected biomarkers in healthy controls. Contaminated cultures will be considered negative. In immunoassays, samples < LLOQ or >HLOQ will be assigned a value below and above the limits of quantification, respectively, through a uniform distribution.

For peer review only

An α-significance level of 0.05 will be used. In single-marker analysis, p-values and CIs will be adjusted for family-wise error rate (FWER) through permutation or false discovery rate (FDR) through Benjamin-Rocheberg, depending on the analysis[118].

Sample Size Justification

We plan to enroll 900 patients to obtain 30 patients with confirmed bacterial disease based on expectations that ~3% of patients will have a positive bacterial culture for Objective 1. Also, we anticipate that 30 patients will meet both viral and malarial definitive etiologies, respectively. If necessary, we will randomly sample patients from the viral and malarial groups. The choice of 30 patients with and 60 patients without bacterial disease was based on feasibility considerations, as was the choice of 20 healthy controls and 160 patients in the analysis of the "probable" diagnosis categories (a random stratified sample from all available subjects). However, with ~90 patients, we could previously identify accurate signatures. We anticipate that in Objective 1, accuracy of previously identified signatures will be estimated with relatively narrow CIs (Table 1). For healthy controls, studying 20 patients will yield a 0.18 CI range for a CV of 0.20 with 0.80 assurance[119]. Analysis of Objective 2 will include a larger number of patients than Objective 1 and shall yield precise confidence intervals and more stable cross-validation based estimates.

For peer review only

Ethics Approval: Ethics approval has been obtained from the Gambia Government/MRCG Joint Ethics Committee (protocol 1616), and the Institutional Review Board of Boston University Medical Center (STUDY00000958).

Protocol registration

The study is registered in clinicaltrials.gov (Protocol number H-38462).

Author contributions

The study concept and design were conceived by CV, GM, and PH with contributions of all other co-authors. Participant enrollment and follow-up will be conducted by YAO and YSI. Sample processing and diagnostic laboratory assays will be supervised by RS, and data management by SG. All on the ground operations in the Gambia will be directly supervised by GM and overseen by CV. QB will be one of the clinicians assigning participants to one of the probable diagnostic groups. The multiplex immunoassay to quantify proteins will be conducted by EK with inputs from MM and CV. Analysis will be performed by CV. CV prepared the first draft of the manuscript. All authors provided edits and reviewed the manuscript providing substantial intellectual contributions.

Funding

This study will be supported by the National Institutes of Health (NIH) of the United States (R21AI140258) and will leverage resources from the project entitled “Will the ongoing use of a two-dose, rather than three-dose schedule of pneumococcal conjugate vaccine, have similar impact in rural Gambia” study funded by the Joint Global Health Trials scheme (MRC UK, Welcome, UK AID, UK National Institute for Health Research) and the Bill and Melinda Gates Foundation.

For peer review only

Competing Interests

None declared

Acknowledgement

We thank the contributions of the clinical, laboratory, and data management staff of MRCG to the final version of this study protocol. Furthermore, we are grateful to Dr. Terrie Taylor for her invaluable support, edits, and guidance to this work.

Data Sharing

Data from this study will be available to study investigators and also to the scientific community under request.

For peer review only

Figure 1 – Definitive and probable diagnostic classification criteria (gold standard of diagnostic signature aim) to assign patients to the malaria (MA), bacterial, (BA) and viral (VI) groups (definitive composite criteria) and to the BA and non-bacterial (NBA) groups.

Figure 2 – Exploratory primary (A) and secondary (B) prognosis outcomes.

For peer review only

Table 1. Precision (exact 95% confidence intervals [CI]) of sensitivity (sample size of 30) and specificity (sample size of 60) when evaluating diagnostic biomarker signatures in analysis of Aim 1.

Proportion	95% CI	
	Sensitivity	Specificity
85%	65-94%	73-95%
90%	73-98%	79-90%
95%	78-99%	86-99%

For peer review only

REFERENCES

1. Nair H, Simoes EA, Rudan I, et al. Global and regional burden of hospital admissions for severe acute lower respiratory infections in young children in 2010: a systematic analysis. *Lancet* 2013;381(9875):1380-90. doi: 10.1016/S0140-6736(12)61901-1
2. Tsao TC, Tsai HC, Chang SC. Clinical Usefulness of Urinary Fatty Acid Binding Proteins in Assessing the Severity and Predicting Treatment Response of Pneumonia in Critically Ill Patients: A Cross-Sectional Study. *Medicine* 2016;95(19):e3682. doi: 10.1097/MD.0000000000003682
3. Engelmann I, Dubos F, Lobert PE, et al. Diagnosis of viral infections using myxovirus resistance protein A (MxA). *Pediatrics* 2015;135(4):e985-93. doi: 10.1542/peds.2014-1946
4. van Houten CB, de Groot JAH, Klein A, et al. A host-protein based assay to differentiate between bacterial and viral infections in preschool children (OPPORTUNITY): a double-blind, multicentre, validation study. *Lancet Infect Dis* 2017;17(4):431-40. doi: 10.1016/S1473-3099(16)30519-9
5. Spoorenberg SM, Vestjens SM, Rijkers GT, et al. YKL-40, CCL18 and SP-D predict mortality in patients hospitalized with community-acquired pneumonia. *Respirology* 2017;22(3):542-50. doi: 10.1111/resp.12924
6. Eden E, Srugo I, Gottlieb T, et al. Diagnostic accuracy of a TRAIL, IP-10 and CRP combination for discriminating bacterial and viral etiologies at the Emergency Department. *The Journal of infection* 2016;73(2):177-80. doi: 10.1016/j.jinf.2016.05.002
7. Relster MM, Holm A, Pedersen C. Plasma cytokines eotaxin, MIP-1alpha, MCP-4, and vascular endothelial growth factor in acute lower respiratory tract infection. *APMIS : acta pathologica, microbiologica, et immunologica Scandinavica* 2017;125(2):148-56. doi: 10.1111/apm.12636
8. Higdon MM, Le T, O'Brien KL, et al. Association of C-Reactive Protein With Bacterial and Respiratory Syncytial Virus-Associated Pneumonia Among Children Aged <5 Years in the PERCH Study. *Clinical infectious diseases : an official publication of the Infectious Diseases Society of America* 2017;64(suppl_3):S378-S86. doi: 10.1093/cid/cix150
9. Yan ST, Sun LC, Jia HB, et al. Procalcitonin levels in bloodstream infections caused by different sources and species of bacteria. *The American journal of emergency medicine* 2017;35(4):579-83. doi: 10.1016/j.ajem.2016.12.017
10. McCluskey SM, Schuetz P, Abers MS, et al. Serial Procalcitonin as a Predictor of Bacteremia and Need for Intensive Care Unit Care in Adults With Pneumonia, Including Those With Highest Severity: A Prospective Cohort Study. *Open forum infectious diseases* 2017;4(1):ofw238. doi: 10.1093/ofid/ofw238
11. Mahende C, Ngasala B, Lusingu J, et al. Profile of C-reactive protein, white cells and neutrophil populations in febrile children from rural north-eastern Tanzania. *The Pan African medical journal* 2017;26:51. doi: 10.11604/pamj.2017.26.51.10264
12. Li YL, Zhai LC, Ji JH, et al. Detection of combined procalcitonin and c-reactive protein applied in the diagnosis of bacterial infections. *Journal of biological regulators and homeostatic agents* 2017;31(1):177-81.
13. Pilotto A, Dini S, Daragjati J, et al. Combined use of the multidimensional prognostic index (MPI) and procalcitonin serum levels in predicting 1-month mortality risk in older patients hospitalized with community-acquired pneumonia (CAP): a prospective study. *Aging clinical and experimental research* 2017 doi: 10.1007/s40520-017-0759-y
14. Andersen SB, Baunbaek Egelund G, Jensen AV, et al. Failure of CRP decline within three days of hospitalization is associated with poor prognosis of Community-acquired Pneumonia. *Infectious diseases* 2017;49(4):251-60. doi: 10.1080/23744235.2016.1253860

For peer review only

15. Chang PY, Tsao SM, Chang JH, et al. Plasma levels of soluble intercellular adhesion molecule-1 as a biomarker for disease severity of patients with community-acquired pneumonia. *Clinica chimica acta; international journal of clinical chemistry* 2016;463:174-80. doi: 10.1016/j.cca.2016.10.030
16. Banoei MM, Vogel HJ, Weljie AM, et al. Plasma metabolomics for the diagnosis and prognosis of H1N1 influenza pneumonia. *Critical care* 2017;21(1):97. doi: 10.1186/s13054-017-1672-7
17. Frenzen FS, Kutschan U, Meiswinkel N, et al. Admission lactate predicts poor prognosis independently of the CRB/CURB-65 scores in community-acquired pneumonia. *Clinical microbiology and infection : the official publication of the European Society of Clinical Microbiology and Infectious Diseases* 2017 doi: 10.1016/j.cmi.2017.07.007
18. Chkhaidze I, Kapanadze N. Cytokines as the Predictors of Severe Mycoplasma Pneumoniae Pneumonia in Children (Review). *Georgian medical news* 2017(267):89-95.
19. Zar HJ, Andronikou S, Nicol MP. Advances in the diagnosis of pneumonia in children. *Bmj* 2017;358:j2739. doi: 10.1136/bmj.j2739
20. Zhou JM, Ye Q. Utility of Assessing Cytokine Levels for the Differential Diagnosis of Pneumonia in a Pediatric Population. *Pediatric critical care medicine : a journal of the Society of Critical Care Medicine and the World Federation of Pediatric Intensive and Critical Care Societies* 2017;18(4):e162-e66. doi: 10.1097/PCC.0000000000001092
21. Curbelo J, Luquero Bueno S, Galvan-Roman JM, et al. Inflammation biomarkers in blood as mortality predictors in community-acquired pneumonia admitted patients: Importance of comparison with neutrophil count percentage or neutrophil-lymphocyte ratio. *PloS one* 2017;12(3):e0173947. doi: 10.1371/journal.pone.0173947
22. Salluh JIF, Souza-Dantas VC, Povoa P. The current status of biomarkers for the diagnosis of nosocomial pneumonias. *Current opinion in critical care* 2017 doi: 10.1097/MCC.0000000000000442
23. Bolatkale M, Duger M, Ulfer G, et al. A novel biochemical marker for community-acquired pneumonia: Ischemia-modified albumin. *The American journal of emergency medicine* 2017;35(8):1121-25. doi: 10.1016/j.ajem.2017.03.018
24. Esposito S, Di Gangi M, Cardinale F, et al. Sensitivity and Specificity of Soluble Triggering Receptor Expressed on Myeloid Cells-1, Midregional Proatrial Natriuretic Peptide and Midregional Proadrenomedullin for Distinguishing Etiology and to Assess Severity in Community-Acquired Pneumonia. *PLoS One* 2016;11(11):e0163262. doi: 10.1371/journal.pone.0163262 [published Online First: 2016/11/16]
25. Li J, Sun L, Xu F, et al. Screening and Identification of APOC1 as a Novel Potential Biomarker for Differentiate of Mycoplasma pneumoniae in Children. *Frontiers in microbiology* 2016;7:1961. doi: 10.3389/fmicb.2016.01961
26. Saghafian-Hedengren S, Mathew JL, Hagel E, et al. Assessment of Cytokine and Chemokine Signatures as Potential Biomarkers of Childhood Community-acquired Pneumonia Severity: A Nested Cohort Study in India. *The Pediatric infectious disease journal* 2017;36(1):102-08. doi: 10.1097/INF.0000000000001364
27. Vogeli A, Ottiger M, Meier MA, et al. Admission levels of asymmetric and symmetric dimethylarginine predict long-term outcome in patients with community-acquired pneumonia. *Respiratory research* 2017;18(1):25. doi: 10.1186/s12931-017-0502-4
28. Vestjens SMT, Spoorenberg SMC, Rijkers GT, et al. High-sensitivity cardiac troponin T predicts mortality after hospitalization for community-acquired pneumonia. *Respirology* 2017;22(5):1000-06. doi: 10.1111/resp.12996
29. Esposito S, Bianchini S, Gambino M, et al. Measurement of lipocalin-2 and syndecan-4 levels to differentiate bacterial from viral infection in children with community-acquired pneumonia. *BMC Pulm Med* 2016;16(1):103. doi: 10.1186/s12890-016-0267-4 [published Online First: 2016/07/22]

For peer review only

30. Loonen AJ, Kesarsing C, Kusters R, et al. High pneumococcal DNA load, procalcitonin and suPAR levels correlate to severe disease development in patients with pneumococcal pneumonia. *European journal of clinical microbiology & infectious diseases : official publication of the European Society of Clinical Microbiology* 2017 doi: 10.1007/s10096-017-2963-2
31. Meier MA, Ottiger M, Vogeli A, et al. Activation of the tryptophan/serotonin pathway is associated with severity and predicts outcomes in pneumonia: results of a long-term cohort study. *Clinical chemistry and laboratory medicine* 2017;55(7):1060-69. doi: 10.1515/cclm-2016-0912
32. Zhang Y, Zhou Y, Li S, et al. The Clinical Characteristics and Predictors of Refractory Mycoplasma pneumoniae Pneumonia in Children. *PloS one* 2016;11(5):e0156465. doi: 10.1371/journal.pone.0156465
33. Levy C, Biscardi S, Dommergues MA, et al. Impact of PCV13 on community-acquired pneumonia by C-reactive protein and procalcitonin levels in children. *Vaccine* 2017 doi: 10.1016/j.vaccine.2017.06.057
34. Kim MW, Lim JY, Oh SH. Mortality prediction using serum biomarkers and various clinical risk scales in community-acquired pneumonia. *Scandinavian journal of clinical and laboratory investigation* 2017:1-7. doi: 10.1080/00365513.2017.1344298
35. Stockmann C, Ampofo K, Killpack J, et al. Procalcitonin Accurately Identifies Hospitalized Children With Low Risk of Bacterial Community-Acquired Pneumonia. *Journal of the Pediatric Infectious Diseases Society* 2017 doi: 10.1093/jpids/piw091
36. Irwin AD, Grant A, Williams R, et al. Predicting Risk of Serious Bacterial Infections in Febrile Children in the Emergency Department. *Pediatrics* 2017;140(2) doi: 10.1542/peds.2016-2853
37. Michelow IC, Olsen K, Lozano J, et al. Epidemiology and clinical characteristics of community-acquired pneumonia in hospitalized children. *Pediatrics* 2004;113(4):701-7.
38. English M, Esamai F, Wasunna A, et al. Assessment of inpatient paediatric care in first referral level hospitals in 13 districts in Kenya. *Lancet* 2004;363(9425):1948-53. doi: 10.1016/S0140-6736(04)16408-8
39. Reyburn H, Mwakasungula E, Chonya S, et al. Clinical assessment and treatment in paediatric wards in the north-east of the United Republic of Tanzania. *Bull World Health Organ* 2008;86(2):132-9.
40. Mackenzie G. The definition and classification of pneumonia. *Pneumonia* 2016;8:14. doi: 10.1186/s41479-016-0012-z
41. Donnelly JP, Baddley JW, Wang HE. Antibiotic utilization for acute respiratory tract infections in u.s. Emergency departments. *Antimicrob Agents Chemother* 2014;58(3):1451-7. doi: 10.1128/AAC.02039-13
42. Johansson EW, Selling KE, Nsona H, et al. Integrated paediatric fever management and antibiotic over-treatment in Malawi health facilities: data mining a national facility census. *Malaria journal* 2016;15(1):396. doi: 10.1186/s12936-016-1439-7
43. Ashu EE, Jarju S, Dione M, et al. Population structure, epidemiology and antibiotic resistance patterns of Streptococcus pneumoniae serotype 5: prior to PCV-13 vaccine introduction in Eastern Gambia. *BMC infectious diseases* 2016;16:33. doi: 10.1186/s12879-016-1370-0
44. Makoka MH, Miller WC, Hoffman IF, et al. Bacterial infections in Lilongwe, Malawi: aetiology and antibiotic resistance. *BMC infectious diseases* 2012;12:67. doi: 10.1186/1471-2334-12-67
45. Feasey NA, Masesa C, Jassi C, et al. Three Epidemics of Invasive Multidrug-Resistant Salmonella Bloodstream Infection in Blantyre, Malawi, 1998-2014. *Clinical infectious diseases : an official publication of the Infectious Diseases Society of America* 2015;61 Suppl 4:S363-71. doi: 10.1093/cid/civ691

For peer review only

46. Gwer S, Newton CR, Berkley JA. Over-diagnosis and co-morbidity of severe malaria in African children: a guide for clinicians. *Am J Trop Med Hyg* 2007;77(6 Suppl):6-13.
47. Bassat Q, Machevo S, O'Callaghan-Gordo C, et al. Distinguishing malaria from severe pneumonia among hospitalized children who fulfilled integrated management of childhood illness criteria for both diseases: a hospital-based study in Mozambique. *Am J Trop Med Hyg* 2011;85(4):626-34. doi: 10.4269/ajtmh.2011.11-0223
48. Ramirez M, Melo-Cristino J. Expanding the diagnosis of pediatric bacteremic pneumococcal pneumonia from blood cultures to molecular methods: advantages and caveats. *Clin Infect Dis* 2010;51(9):1050-2. doi: 10.1086/656580
49. Bradley JS, Byington CL, Shah SS, et al. The management of community-acquired pneumonia in infants and children older than 3 months of age: clinical practice guidelines by the Pediatric Infectious Diseases Society and the Infectious Diseases Society of America. *Clin Infect Dis* 2011;53(7):e25-76. doi: 10.1093/cid/cir531
50. Cherian T, Mulholland EK, Carlin JB, et al. Standardized interpretation of paediatric chest radiographs for the diagnosis of pneumonia in epidemiological studies. *Bull World Health Organ* 2005;83(5):353-9. doi: /S0042-96862005000500011
51. Edwards M, Lawson Z, Morris S, et al. The presence of radiological features on chest radiographs: how well do clinicians agree? *Clin Radiol* 2012;67(7):664-8. doi: 10.1016/j.crad.2011.12.003
52. Davies HD, Wang EE, Manson D, et al. Reliability of the chest radiograph in the diagnosis of lower respiratory infections in young children. *Pediatr Infect Dis J* 1996;15(7):600-4.
53. Albaum MN, Hill LC, Murphy M, et al. Interobserver reliability of the chest radiograph in community-acquired pneumonia. PORT Investigators. *Chest* 1996;110(2):343-50.
54. Hagaman JT, Rouan GW, Shipley RT, et al. Admission chest radiograph lacks sensitivity in the diagnosis of community-acquired pneumonia. *Am J Med Sci* 2009;337(4):236-40. doi: 10.1097/MAJ.0b013e31818ad805
55. Johnson J, Kline JA. Intraobserver and interobserver agreement of the interpretation of pediatric chest radiographs. *Emerg Radiol* 2010;17(4):285-90. doi: 10.1007/s10140-009-0854-2
56. Loeb MB, Carusone SB, Marrie TJ, et al. Interobserver reliability of radiologists' interpretations of mobile chest radiographs for nursing home-acquired pneumonia. *J Am Med Dir Assoc* 2006;7(7):416-9. doi: 10.1016/j.jamda.2006.02.004
57. O'Grady KA, Torzillo PJ, Ruben AR, et al. Identification of radiological alveolar pneumonia in children with high rates of hospitalized respiratory infections: comparison of WHO-defined and pediatric pulmonologist diagnosis in the clinical context. *Pediatr Pulmonol* 2012;47(4):386-92. doi: 10.1002/ppul.21551
58. Murdoch DR, O'Brien KL, Scott JA, et al. Breathing new life into pneumonia diagnostics. *J Clin Microbiol* 2009;47(11):3405-8. doi: 10.1128/JCM.01685-09
59. Gonsalves WI, Cornish N, Moore M, et al. Effects of volume and site of blood draw on blood culture results. *J Clin Microbiol* 2009;47(11):3482-5. doi: 10.1128/JCM.02107-08
60. Avni T, Mansur N, Leibovici L, et al. PCR using blood for diagnosis of invasive pneumococcal disease: systematic review and meta-analysis. *J Clin Microbiol* 2010;48(2):489-96. doi: 10.1128/JCM.01636-09
61. Resti M, Moriondo M, Cortimiglia M, et al. Community-acquired bacteremic pneumococcal pneumonia in children: diagnosis and serotyping by real-time polymerase chain reaction using blood samples. *Clin Infect Dis* 2010;51(9):1042-9. doi: 10.1086/656579
62. Morpeth SC, Deloria Knoll M, Scott JAG, et al. Detection of Pneumococcal DNA in Blood by Polymerase Chain Reaction for Diagnosing Pneumococcal Pneumonia in Young Children From Low- and Middle-Income Countries. *Clinical infectious diseases : an official publication of the Infectious Diseases Society of America* 2017;64(suppl_3):S347-S56. doi: 10.1093/cid/cix145

For peer review only

63. Anjay MA, Anoop P. Diagnostic utility of rapid immunochromatographic urine antigen testing in suspected pneumococcal infections. *Archives of disease in childhood* 2008;93(7):628-31. doi: 10.1136/adc.2008.138958
64. Charkaluk ML, Kalach N, Mvogo H, et al. Assessment of a rapid urinary antigen detection by an immunochromatographic test for diagnosis of pneumococcal infection in children. *Diagn Microbiol Infect Dis* 2006;55(2):89-94. doi: 10.1016/j.diagmicrobio.2005.10.013
65. Thea DM, Seidenberg P, Park DE, et al. Limited Utility of Polymerase Chain Reaction in Induced Sputum Specimens for Determining the Causes of Childhood Pneumonia in Resource-Poor Settings: Findings From the Pneumonia Etiology Research for Child Health (PERCH) Study. *Clinical infectious diseases : an official publication of the Infectious Diseases Society of America* 2017;64(suppl_3):S289-S300. doi: 10.1093/cid/cix098
66. Murdoch DR, Morpeth SC, Hammitt LL, et al. The Diagnostic Utility of Induced Sputum Microscopy and Culture in Childhood Pneumonia. *Clinical infectious diseases : an official publication of the Infectious Diseases Society of America* 2017;64(suppl_3):S280-S88. doi: 10.1093/cid/cix090
67. Koram KA, Molyneux ME. When is "malaria" malaria? The different burdens of malaria infecttion, malaria disease, and malaria-like illnesses. . *Am J Trop Med Hyg* 2007;77(Suppl 6):1-5.
68. D'Acremont V, Kilowoko M, Kyungu E, et al. Beyond malaria--causes of fever in outpatient Tanzanian children. *N Engl J Med* 2014;370(9):809-17. doi: 10.1056/NEJMoa1214482
69. Maniaci V, Dauber A, Weiss S, et al. Procalcitonin in young febrile infants for the detection of serious bacterial infections. *Pediatrics* 2008;122(4):701-10. doi: 10.1542/peds.2007-3503
70. Nascimento-Carvalho CM, Cardoso MR, Barral A, et al. Procalcitonin is useful in identifying bacteraemia among children with pneumonia. *Scandinavian journal of infectious diseases* 2010;42(9):644-9. doi: 10.3109/00365541003796775
71. Zhydkov A, Christ-Crain M, Thomann R, et al. Utility of procalcitonin, C-reactive protein and white blood cells alone and in combination for the prediction of clinical outcomes in community-acquired pneumonia. *Clinical chemistry and laboratory medicine* 2015;53(4):559-66. doi: 10.1515/cclm-2014-0456
72. Tang BM, Eslick GD, Craig JC, et al. Accuracy of procalcitonin for sepsis diagnosis in critically ill patients: systematic review and meta-analysis. *Lancet Infect Dis* 2007;7(3):210-7. doi: 10.1016/S1473-3099(07)70052-X
73. Thayyil S, Shenoy M, Hamaluba M, et al. Is procalcitonin useful in early diagnosis of serious bacterial infections in children? *Acta Paediatr* 2005;94(2):155-8.
74. Kruger S, Ewig S, Marre R, et al. Procalcitonin predicts patients at low risk of death from community-acquired pneumonia across all CRB-65 classes. *Eur Respir J* 2008;31(2):349-55. doi: 10.1183/09031936.00054507
75. Hirakata Y, Yanagihara K, Kurihara S, et al. Comparison of usefulness of plasma procalcitonin and C-reactive protein measurements for estimation of severity in adults with community-acquired pneumonia. *Diagnostic microbiology and infectious disease* 2008;61(2):170-4. doi: 10.1016/j.diagmicrobio.2008.01.014
76. Meili M, Kutz A, Briel M, et al. Infection biomarkers in primary care patients with acute respiratory tract infections-comparison of Procalcitonin and C-reactive protein. *BMC pulmonary medicine* 2016;16:43. doi: 10.1186/s12890-016-0206-4
77. Lindstrom ST, Wong EK. Procalcitonin, a valuable biomarker assisting clinical decision-making in the management of community-acquired pneumonia. *Internal medicine journal* 2014;44(4):390-7. doi: 10.1111/imj.12374

For peer review only

78. Heiskanen-Kosma T, Korppi M. Serum C-reactive protein cannot differentiate bacterial and viral aetiology of community-acquired pneumonia in children in primary healthcare settings. *Scand J Infect Dis* 2000;32(4):399-402.
79. Korppi M, Remes S, Heiskanen-Kosma T. Serum procalcitonin concentrations in bacterial pneumonia in children: a negative result in primary healthcare settings. *Pediatr Pulmonol* 2003;35(1):56-61. doi: 10.1002/ppul.10201
80. Rodriguez AH, Aviles-Jurado FX, Diaz E, et al. Procalcitonin (PCT) levels for ruling-out bacterial coinfection in ICU patients with influenza: A CHAID decision-tree analysis. *The Journal of infection* 2016;72(2):143-51. doi: 10.1016/j.jinf.2015.11.007
81. Haran JP, Beaudoin FL, Suner S, et al. C-reactive protein as predictor of bacterial infection among patients with an influenza-like illness. *Am J Emerg Med* 2013;31(1):137-44. doi: 10.1016/j.ajem.2012.06.026
82. Johansson N, Kalin M, Backman-Johansson C, et al. Procalcitonin levels in community-acquired pneumonia - correlation with aetiology and severity. *Scand J Infect Dis* 2014;46(11):787-91. doi: 10.3109/00365548.2014.945955
83. Schuetz P, Batschwaroff M, Dusemund F, et al. Effectiveness of a procalcitonin algorithm to guide antibiotic therapy in respiratory tract infections outside of study conditions: a post-study survey. *European journal of clinical microbiology & infectious diseases : official publication of the European Society of Clinical Microbiology* 2010;29(3):269-77. doi: 10.1007/s10096-009-0851-0
84. Long W, Deng X, Zhang Y, et al. Procalcitonin guidance for reduction of antibiotic use in low-risk outpatients with community-acquired pneumonia. *Respirology* 2011;16(5):819-24. doi: 10.1111/j.1440-1843.2011.01978.x
85. Christ-Crain M, Stolz D, Bingisser R, et al. Procalcitonin guidance of antibiotic therapy in community-acquired pneumonia: a randomized trial. *American journal of respiratory and critical care medicine* 2006;174(1):84-93. doi: 10.1164/rccm.200512-1922OC
86. Huang DT, Weissfeld LA, Kellum JA, et al. Risk prediction with procalcitonin and clinical rules in community-acquired pneumonia. *Annals of emergency medicine* 2008;52(1):48-58 e2. doi: 10.1016/j.annemergmed.2008.01.003
87. Don M, Valent F, Korppi M, et al. Efficacy of serum procalcitonin in evaluating severity of community-acquired pneumonia in childhood. *Scand J Infect Dis* 2007;39(2):129-37. doi: 10.1080/00365540600951283
88. Michelow IC, Katz K, McCracken GH, et al. Systemic cytokine profile in children with community-acquired pneumonia. *Pediatric pulmonology* 2007;42(7):640-5. doi: 10.1002/ppul.20633
89. Florin TA, Ambroggio L. Biomarkers for community-acquired pneumonia in the emergency department. *Curr Infect Dis Rep* 2014;16(12):451. doi: 10.1007/s11908-014-0451-8
90. Muller B, Harbarth S, Stolz D, et al. Diagnostic and prognostic accuracy of clinical and laboratory parameters in community-acquired pneumonia. *BMC Infect Dis* 2007;7:10. doi: 10.1186/1471-2334-7-10
91. Bafadhel M, Clark TW, Reid C, et al. Procalcitonin and C-reactive protein in hospitalized adult patients with community-acquired pneumonia or exacerbation of asthma or COPD. *Chest* 2011;139(6):1410-18. doi: 10.1378/chest.10-1747
92. Principi N, Esposito S. Biomarkers in Pediatric Community-Acquired Pneumonia. *International journal of molecular sciences* 2017;18(2) doi: 10.3390/ijms18020447
93. Bordon J, Aliberti S, Fernandez-Botran R, et al. Understanding the roles of cytokines and neutrophil activity and neutrophil apoptosis in the protective versus deleterious inflammatory response in pneumonia. *International journal of infectious diseases : IJID : official publication of the International Society for Infectious Diseases* 2013;17(2):e76-83. doi: 10.1016/j.ijid.2012.06.006

For peer review only

94. Mahajan P, Kuppermann N, Mejias A, et al. Association of RNA Biosignatures With Bacterial Infections in Febrile Infants Aged 60 Days or Younger. *JAMA* 2016;316(8):846-57. doi: 10.1001/jama.2016.9207 [published Online First: 2016/08/24]
95. Herberg JA, Kaforou M, Wright VJ, et al. Diagnostic Test Accuracy of a 2-Transcript Host RNA Signature for Discriminating Bacterial vs Viral Infection in Febrile Children. *JAMA* 2016;316(8):835-45. doi: 10.1001/jama.2016.11236 [published Online First: 2016/08/24]
96. Huang H, Ideh RC, Gitau E, et al. Discovery and Validation of Biomarkers to Guide Clinical Management of Pneumonia in African Children. *Clin Infect Dis* 2014 doi: 10.1093/cid/ciu202
97. Oved K, Cohen A, Boico O, et al. A novel host-proteome signature for distinguishing between acute bacterial and viral infections. *PLoS One* 2015;10(3):e0120012. doi: 10.1371/journal.pone.0120012 [published Online First: 2015/03/19]
98. Srugo I, Klein A, Stein M, et al. Validation of a Novel Assay to Distinguish Bacterial and Viral Infections. *Pediatrics* 2017;140(4) doi: 10.1542/peds.2016-3453 [published Online First: 2017/09/15]
99. Stein M, Lipman-Arens S, Oved K, et al. A novel host-protein assay outperforms routine parameters for distinguishing between bacterial and viral lower respiratory tract infections. *Diagn Microbiol Infect Dis* 2018;90(3):206-13. doi: 10.1016/j.diagmicrobio.2017.11.011 [published Online First: 2017/12/24]
100. Ashkenazi-Hoffnung L, Oved K, Navon R, et al. A host-protein signature is superior to other biomarkers for differentiating between bacterial and viral disease in patients with respiratory infection and fever without source: a prospective observational study. *Eur J Clin Microbiol Infect Dis* 2018;37(7):1361-71. doi: 10.1007/s10096-018-3261-3 [published Online First: 2018/04/28]
101. Self WH, Rosen J, Sharp SC, et al. Diagnostic Accuracy of FebriDx: A Rapid Test to Detect Immune Responses to Viral and Bacterial Upper Respiratory Infections. *J Clin Med* 2017;6(10) doi: 10.3390/jcm6100094 [published Online First: 2017/10/11]
102. Shapiro NI, Self WH, Rosen J, et al. A prospective, multi-centre US clinical trial to determine accuracy of FebriDx point-of-care testing for acute upper respiratory infections with and without a confirmed fever. *Ann Med* 2018;50(5):420-29. doi: 10.1080/07853890.2018.1474002 [published Online First: 2018/05/19]
103. Dittrich S, Tadesse BT, Moussy F, et al. Target Product Profile for a Diagnostic Assay to Differentiate between Bacterial and Non-Bacterial Infections and Reduce Antimicrobial Overuse in Resource-Limited Settings: An Expert Consensus. *PLoS One* 2016;11(8):e0161721. doi: 10.1371/journal.pone.0161721 [published Online First: 2016/08/26]
104. Valim C, Ahmad R, Lanaspa M, et al. Responses to Bacteria, Virus, and Malaria Distinguish the Etiology of Pediatric Clinical Pneumonia. *Am J Respir Crit Care Med* 2016;193(4):448-59. doi: 10.1164/rccm.201506-1100OC [published Online First: 2015/10/16]
105. Irwin AD, Marriage F, Mankhambo LA, et al. Novel biomarker combination improves the diagnosis of serious bacterial infections in Malawian children. *BMC Med Genomics* 2012;5:13. doi: 10.1186/1755-8794-5-13 [published Online First: 2012/05/09]
106. Berlutti F, Pantanella F, Natalizi T, et al. Antiviral properties of lactoferrin--a natural immunity molecule. *Molecules* 2011;16(8):6992-7018. doi: 10.3390/molecules16086992 [published Online First: 2011/08/19]
107. Bertens LC, Broekhuizen BD, Naaktgeboren CA, et al. Use of expert panels to define the reference standard in diagnostic research: a systematic review of published methods and reporting. *PLoS medicine* 2013;10(10):e1001531. doi: 10.1371/journal.pmed.1001531

For peer review only

108. Reitsma JB, Rutjes AW, Khan KS, et al. A review of solutions for diagnostic accuracy studies with an imperfect or missing reference standard. *Journal of clinical epidemiology* 2009;62(8):797-806. doi: 10.1016/j.jclinepi.2009.02.005
109. Howie SR. Blood sample volumes in child health research: review of safe limits. *Bull World Health Organ* 2011;89(1):46-53. doi: 10.2471/BLT.10.080010
110. Bates DW, Lee TH. Rapid classification of positive blood cultures. Prospective validation of a multivariate algorithm. *JAMA* 1992;267(14):1962-6. [published Online First: 1992/04/08]
111. Adegbola RA, Falade AG, Sam BE, et al. The etiology of pneumonia in malnourished and well-nourished Gambian children. *The Pediatric infectious disease journal* 1994;13(11):975-82.
112. Gunson RN, Collins TC, Carman WF. Real-time RT-PCR detection of 12 respiratory viral infections in four triplex reactions. *J Clin Virol* 2005;33(4):341-4. doi: 10.1016/j.jcv.2004.11.025 [published Online First: 2005/06/02]
113. Scholman RC, Giovannone B, Hiddingh S, et al. Effect of anticoagulants on 162 circulating immune related proteins in healthy subjects. *Cytokine* 2018;106:114-24. doi: 10.1016/j.cyto.2017.10.021 [published Online First: 2017/11/02]
114. Valim C, Ahmad R, Lanaspa M, et al. Responses to Bacteria, Virus and Malaria Distinguish the Etiology of Pediatric Clinical Pneumonia. *Am J Respir Crit Care Med* 2015 doi: 10.1164/rccm.201506-1100OC
115. DeLong ER, DeLong DM, Clarke-Pearson DL. Comparing the areas under two or more correlated receiver operating characteristic curves: a nonparametric approach. *Biometrics* 1988;44(3):837-45.
116. Guyon I, Weston J, Barnhill S, et al. Gene Selection for Cancer Classification using Support Vector Machines. *Machine Learning* 2002;46(1-3):389-422. doi: 10.1023/A:1012487302797
117. Becker N, Toedt G, Lichter P, et al. Elastic SCAD as a novel penalization method for SVM classification tasks in high-dimensional data. *BMC Bioinformatics* 2011;12:138. doi: 10.1186/1471-2105-12-138
118. Goeman JJ, Solari A. Multiple hypothesis testing in genomics. *Stat Med* 2014;33(11):1946-78. doi: 10.1002/sim.6082
119. Kelley K. Sample size planning for the coefficient of variation from the accuracy in parameter estimation approach. *Behavior research methods* 2007;39(4):755-66.

For peer review only

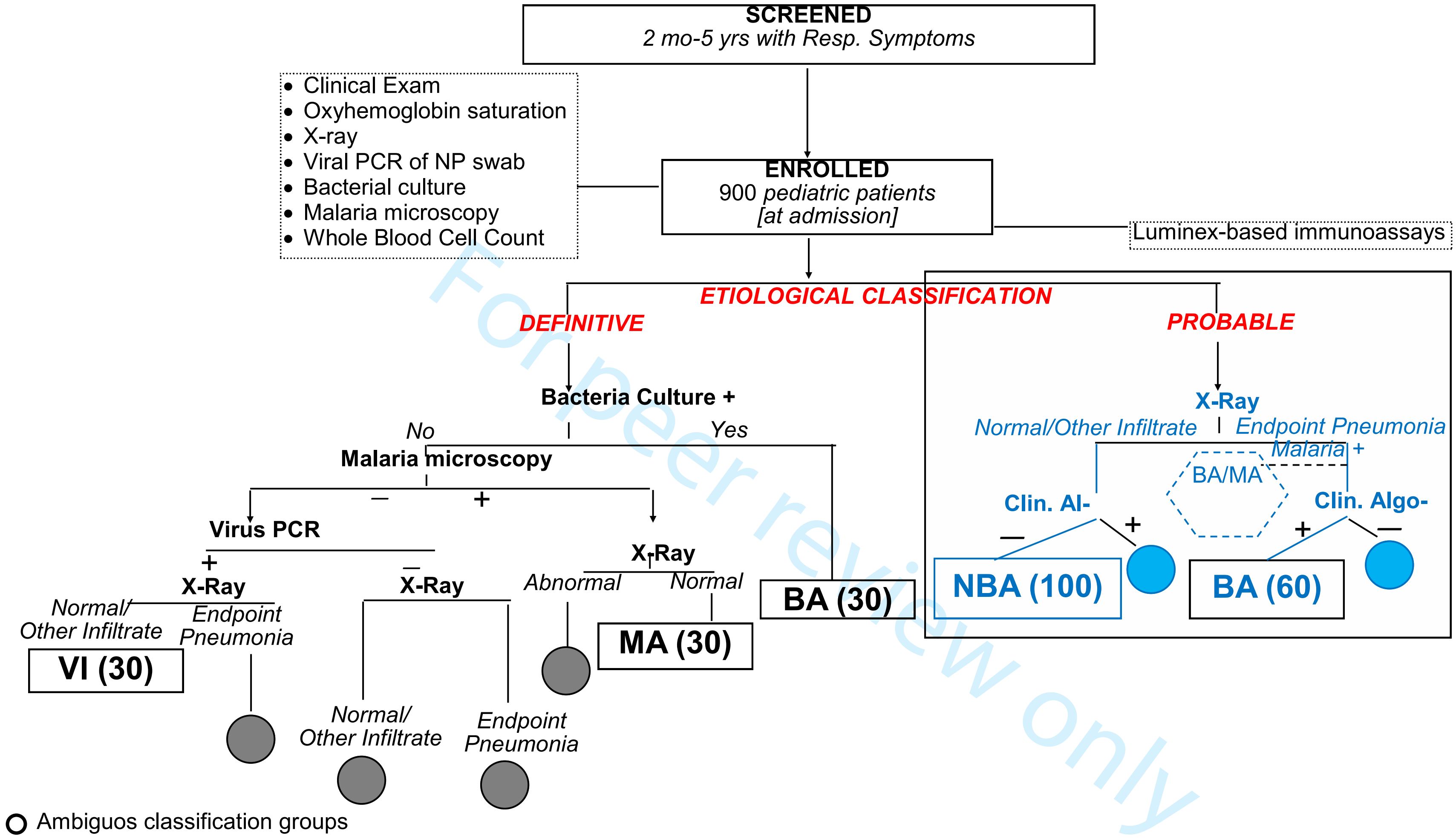

Endpoint pneumonia: consolidation or pleural effusion.

3 PROGNOSIS GROUPS (N = 160)		
**Poor prognosis**	**Adverse progress of disease**	**Good Prognosis**
• Dearth	• Re-admission<br>• Need to switch antibiotic therapy ≤ 48 hrs of hospitalization<br>• > 3 days of hospitalization	• Discharged well<br>• No need to switch antibiotic therapy during admission<br>• < 3 days of hospitaliza-

CONTINUOUS AND CATEGORICAL POOR PROGNOSIS OUTCOMES (N = 160)
• Area under 5-day SaO_2<br>• Need to switch antibiotic therapy ≤ 48 hrs of hospitalization<br>• Length of hospital stay

For peer review only

Section & Topic	No	Item
**TITLE OR ABSTRACT**		
	**1**	Identification as a study of diagnostic accuracy using at least one measure of accuracy (such as sensitivity, specificity, predictive values, or AUC)
**ABSTRACT**		
	**2**	Structured summary of study design, methods, results, and conclusions (for specific guidance, see STARD for Abstracts)
**INTRODUCTION**		
	**3**	Scientific and clinical background, including the intended use and clinical role of the index test
	**4**	Study objectives and hypotheses
**METHODS**		
Study design	**5**	Whether data collection was planned before the index test and reference standard were performed (prospective study) or after (retrospective study)
Participants	**6**	Eligibility criteria
	**7**	On what basis potentially eligible participants were identified (such as symptoms, results from previous tests, inclusion in registry)
	**8**	Where and when potentially eligible participants were identified (setting, location and dates)
	**9**	Whether participants formed a consecutive, random or convenience series
Test methods	**10a**	Index test, in sufficient detail to allow replication
	**10b**	Reference standard, in sufficient detail to allow replication
	**11**	Rationale for choosing the reference standard (if alternatives exist)
	**12a**	Definition of and rationale for test positivity cut-offs or result categories of the index test, distinguishing pre-specified from exploratory
	**12b**	Definition of and rationale for test positivity cut-offs or result categories of the reference standard, distinguishing pre-specified from exploratory
	**13a**	Whether clinical information and reference standard results were available to the performers/readers of the index test
	**13b**	Whether clinical information and index test results were available to the assessors of the reference standard
Analysis	**14**	Methods for estimating or comparing measures of diagnostic accuracy
	**15**	How indeterminate index test or reference standard results were handled
	**16**	How missing data on the index test and reference standard were handled
	**17**	Any analyses of variability in diagnostic accuracy, distinguishing pre-specified from exploratory
	**18**	Intended sample size and how it was determined
**RESULTS**		
Participants	**19**	Flow of participants, using a diagram
	**20**	Baseline demographic and clinical characteristics of participants
	**21a**	Distribution of severity of disease in those with the target condition
	**21b**	Distribution of alternative diagnoses in those without the target condition
	**22**	Time interval and any clinical interventions between index test and reference standard
Test results	**23**	Cross tabulation of the index test results (or their distribution) by the results of the reference standard
	**24**	Estimates of diagnostic accuracy and their precision (such as 95% confidence intervals)
	**25**	Any adverse events from performing the index test or the reference standard
**DISCUSSION**		
	**26**	Study limitations, including sources of potential bias, statistical uncertainty, and generalisability
	**27**	Implications for practice, including the intended use and clinical role of the index test
**OTHER INFORMATION**		
	**28**	Registration number and name of registry
	**29**	Where the full study protocol can be accessed
	**30**	Sources of funding and other support; role of funders

For peer review only

BY SA

STARD 2015

AIM

STARD stands for “Standards for Reporting Diagnostic accuracy studies”. This list of items was developed to contribute to the completeness and transparency of reporting of diagnostic accuracy studies. Authors can use the list to write informative study reports. Editors and peer-reviewers can use it to evaluate whether the information has been included in manuscripts submitted for publication.

EXPLANATION

A **diagnostic accuracy study** evaluates the ability of one or more medical tests to correctly classify study participants as having a **target condition.** This can be a disease, a disease stage, response or benefit from therapy, or an event or condition in the future. A medical test can be an imaging procedure, a laboratory test, elements from history and physical examination, a combination of these, or any other method for collecting information about the current health status of a patient.

The test whose accuracy is evaluated is called **index test.** A study can evaluate the accuracy of one or more index tests. Evaluating the ability of a medical test to correctly classify patients is typically done by comparing the distribution of the index test results with those of the **reference standard**. The reference standard is the best available method for establishing the presence or absence of the target condition. An accuracy study can rely on one or more reference standards.

If test results are categorized as either positive or negative, the cross tabulation of the index test results against those of the reference standard can be used to estimate the **sensitivity** of the index test (the proportion of participants *with* the target condition who have a positive index test), and its **specificity** (the proportion *without* the target condition who have a negative index test). From this cross tabulation (sometimes referred to as the contingency or “2x2” table), several other accuracy statistics can be estimated, such as the positive and negative **predictive values** of the test. Confidence intervals around estimates of accuracy can then be calculated to quantify the statistical **precision** of the measurements.

If the index test results can take more than two values, categorization of test results as positive or negative requires a **test positivity cut-off**. When multiple such cut-offs can be defined, authors can report a receiver operating characteristic (ROC) curve which graphically represents the combination of sensitivity and specificity for each possible test positivity cut-off. The **area under the ROC curve** informs in a single numerical value about the overall diagnostic accuracy of the index test.

The **intended use** of a medical test can be diagnosis, screening, staging, monitoring, surveillance, prediction or prognosis. The **clinical role** of a test explains its position relative to existing tests in the clinical pathway. A replacement test, for example, replaces an existing test. A triage test is used before an existing test; an add-on test is used after an existing test.

Besides diagnostic accuracy, several other outcomes and statistics may be relevant in the evaluation of medical tests. Medical tests can also be used to classify patients for purposes other than diagnosis, such as staging or prognosis. The STARD list was not explicitly developed for these other outcomes, statistics, and study types, although most STARD items would still apply.

DEVELOPMENT

This STARD list was released in 2015. The 30 items were identified by an international expert group of methodologists, researchers, and editors. The guiding principle in the development of STARD was to select items that, when reported, would help readers to judge the potential for bias in the study, to appraise the applicability of the study findings and the validity of conclusions and recommendations. The list represents an update of the first version, which was published in 2003.

More information can be found on http://www.equator-network.org/reporting-guidelines/stard.

For peer review only - http://bmjopen.bmj.com/site/about/guidelines.xhtml

BMJ Open

A Study Protocol for an Observational Study Seeking Diagnostic and Prognostic Biomarkers for Childhood Bacterial Pneumonia in Sub-Saharan Africa

Journal:	*BMJ Open*
Manuscript ID	bmjopen-2020-046590.R1
Article Type:	Protocol
Date Submitted by the Author:	29-Apr-2021
Complete List of Authors:	Valim, Clarissa; Boston University School of Public Health, Department of Global Health<br>Olatunji, Yekin; Medical Research Council The Gambia<br>Isa, Yasir; Medical Research Council The Gambia<br>Salaudeen, Rasheed; Medical Research Council The Gambia<br>Golam, Sarwar; Medical Research Council The Gambia<br>Knol, Edward; University Medical Center Utrecht Department of Rheumatology and Clinical Immunology, Center of Translational Immunology<br>Kanyl, Sheriffo; Bansang Hospital<br>Jammeh, Abdoulle; Basse Hospital<br>Bassat, Quique; ISGlobal, Hospital Clínic - Universitat de Barcelona; Centro de Investigação em Saúde de Manhiça (CISM)<br>deJager, Wilco; University Medical Center Utrecht Department of Rheumatology and Clinical Immunology, Center of Translational Immunology; Luminex Corp<br>Diaz, Alejandro; Harvard Medical School, Department of Medicine; Brigham and Women's Hospital, Division of Pulmonary and Critical Care Medicine<br>Wiegand, Roger; Independent Researcher<br>Ramirez, Julio; University of Louisville, Division of Infectious Diseases<br>Moses, Marsha ; Children's Hospital Boston, Vascular Biology Program; Harvard Medical School, Department of Surgery<br>D'Alessandro, Umberto; Medical Research Council Unit, Disease Elimination and Control; London School of Hygiene and Tropical Medicine,<br>Hibberd, Patricia; Boston University School of Public Health<br>Mackenzie, Grant; Medical Research Council Unit The Gambia at the London School of Hygiene and Tropical Medicine; London School of Hygiene and Tropical Medicine Faculty of Infectious and Tropical Diseases, Department of Disease Control
**Primary Subject Heading**:	Diagnostics
Secondary Subject Heading:	Infectious diseases, Respiratory medicine
Keywords:	Respiratory infections < THORACIC MEDICINE, Diagnostic microbiology < INFECTIOUS DISEASES, Paediatric infectious disease & immunisation < PAEDIATRICS, Paediatric thoracic medicine < PAEDIATRICS

SCHOLARONE™
Manuscripts

I, the Submitting Author has the right to grant and does grant on behalf of all authors of the Work (as defined in the below author licence), an exclusive licence and/or a non-exclusive licence for contributions from authors who are: i) UK Crown employees; ii) where BMJ has agreed a CC-BY licence shall apply, and/or iii) in accordance with the terms applicable for US Federal Government officers or employees acting as part of their official duties; on a worldwide, perpetual, irrevocable, royalty-free basis to BMJ Publishing Group Ltd ("BMJ") its licensees and where the relevant Journal is co-owned by BMJ to the co-owners of the Journal, to publish the Work in this journal and any other BMJ products and to exploit all rights, as set out in our licence.

The Submitting Author accepts and understands that any supply made under these terms is made by BMJ to the Submitting Author unless you are acting as an employee on behalf of your employer or a postgraduate student of an affiliated institution which is paying any applicable article publishing charge ("APC") for Open Access articles. Where the Submitting Author wishes to make the Work available on an Open Access basis (and intends to pay the relevant APC), the terms of reuse of such Open Access shall be governed by a Creative Commons licence – details of these licences and which Creative Commons licence will apply to this Work are set out in our licence referred to above.

Other than as permitted in any relevant BMJ Author's Self Archiving Policies, I confirm this Work has not been accepted for publication elsewhere, is not being considered for publication elsewhere and does not duplicate material already published. I confirm all authors consent to publication of this Work and authorise the granting of this licence.

For peer review only

A Study Protocol for an Observational Study Seeking Diagnostic and Prognostic Biomarkers for Childhood Bacterial Pneumonia in Sub-Saharan Africa

Clarissa Valim[1], Yekin Ajauoi Olatunji[2], Yasir Shitu Isa[2], Rasheed Salaudeen[2], Sarwar Golam[2], Edward F. Knol[3], Sheriffo Kanyi[4], Abdoulie Jammeh[5], Quique Bassat[6-10], Wilco DeJager[3,11], Alejandro A. Diaz[12,13], Roger C. Wiegand[14], Julio A. Ramirez[15], Marsha A. Moses[16,17], Umberto D'Alessandro[2,18], Patricia Hibberd*[1], Grant Mackenzie*[2,18-20]

* These authors equally contributed to this work

AFFILIATIONS

1. Department of Global Health, Boston University School of Public Health, Boston, USA
2. Medical Research Council (MRC) Unit The Gambia at London School of Hygiene & Tropical Medicine, Fajara, The Gambia
3. Center of Translational Immunology, University Medical Center Utrecht, The Netherlands
4. Bansang Hospital, Central River Region, The Gambia
5. Basse Hospital, Upper River Region, The Gambia
6. ISGlobal, Hospital Clínic - Universitat de Barcelona, Barcelona, Spain
7. Centro de Investigação em Saúde de Manhiça (CISM), Maputo, Mozambique
8. ICREA, Pg. Lluís Companys 23, 08010 Barcelona, Spain.
9. Pediatric Infectious Diseases Unit, Pediatrics Department, Hospital Sant Joan de Déu (University of Barcelona), Barcelona, Spain
10. Consorcio de Investigación Biomédica en Red de Epidemiología y Salud Pública (CIBERESP), Madrid, Spain
11. Luminex corporation, Austin, Texas, USA
12. Department of Medicine, Harvard Medical School
13. Division of Pulmonary and Critical Care Medicine, Brigham and Women's Hospital, Harvard University, Boston, Massachusetts, USA
14. Independent Researcher, Boston, Massachusetts, USA

For peer review only - http://bmjopen.bmj.com/site/about/guidelines.xhtml

15. Division of Infectious Diseases, Department of Medicine, University of Louisville School of Medicine, Louisville, Kentucky

16. Vascular Biology Program, Children's Hospital Boston, Massachusetts, USA

17. Department of Surgery, Harvard Medical School, Massachusetts, USA

18. Department of Disease Control, Faculty of Infectious and Tropical Diseases, London School of Hygiene & Tropical Medicine, London, UK

19. Murdoch Children's Research Institute, Melbourne, Australia

20. Department of Paediatrics, University of Melbourne, Australia

Corresponding author:

Clarissa Valim

Dept. of Global Health, Boston University School of Public Health, Boston, Massachusetts, USA

cvalim@bu.edu

Keywords:

Biomarkers, pneumonia, bacterial infection, inflammatory proteins, machine learning

Word Count of the manuscript: 4,056

For peer review only - http://bmjopen.bmj.com/site/about/guidelines.xhtml

Abstract

Introduction:

Clinically diagnosed pneumonia in children is a leading cause of pediatric hospitalization and mortality. The etiology is usually bacterial or viral, but malaria can cause a syndrome indistinguishable from clinical pneumonia. There is no method with high sensitivity to detect a bacterial infection in these patients and, as result, antibiotics are frequently overprescribed. Conversely, unrecognized concomitant bacterial infection in patients with malarial infections occur with omission of antibiotic therapy from patients with bacterial infections.

Previously, we identified two combinations of blood proteins with 96% sensitivity and 86% specificity for detecting bacterial disease. The current project aims to validate and improve these combinations by evaluating additional biomarkers in pediatric patients with clinical pneumonia. Our goal is to describe combinations of a limited number of proteins with high sensitivity and specificity for bacterial infection to be incorporated in future point-of-care tests. Furthermore, we seek to explore signatures to prognosticate clinical pneumonia.

Methods and analysis:

Patients (N = 900) aged 2-59 months presenting with clinical pneumonia at two Gambian hospitals will be enrolled and classified according to criteria for definitive bacterial etiology (based on microbiological tests and chest radiographs). We will measure proteins at admission using Luminex®-based immunoassays in 90 children with definitive and 160 with probable bacterial aetiology, and 160 children classified according to the prognosis of their disease. Previously identified diagnostic signatures will be assessed through accuracy measures. Moreover, we will seek new diagnostic and prognostic signatures through machine learning methods, including support vector machine, penalized regression, and classification trees.

For peer review only - http://bmjopen.bmj.com/site/about/guidelines.xhtml

Ethics and dissemination

Ethics approval has been obtained from: the Gambia Government/MRCG Joint Ethics Committee (protocol 1616), and the Institutional Review Board of Boston University Medical Center (STUDY00000958). Study results will be disseminated to the staff of the study hospitals, in scientific seminars and meetings, and in publications.

For peer review only

Article Summary

Strengths and limitations of this study:

- ✓ This study will be conducted in a resource-limited country for future development of a point-of-care (POC) test for these settings.
- ✓ We will quantify known and recently identified biomarkers that will be combined using different machine learning approaches.
- ✓ Children will be followed for 30 days after discharge to yield mid-term prognostic assessment of the clinical pneumonia.
- ✓ A difficulty shared by studies aiming to identify biomarkers to diagnose bacterial infection is lack of an optimal gold standard for bacterial infection, thus, we will use a composite criteriion based on microbiological and radiological evidence of infection (primary criteria) and probable criteria based on expert evaluation of clinical and laboratory data.
- ✓ Although the sample size is relatively small, results will establish a benchmark against which the identified signatures in populations in other areas and in different subgroups, such as children with undernutrition, can be compared.

For peer review only - http://bmjopen.bmj.com/site/about/guidelines.xhtml

Abbreviation List: AUC-ROC: area under the receiver operating characteristic curve; BA: bacterial group; CI: confidence interval; CRF: case report form; CRP: C-reactive protein; CSF: cerebrospinal fluid; EDTA: ethylenediaminetetraacetic acid; FBC: full blood cell count; FDR: false discovery rate; FWER: family-wise error rate; GCSF: granulocyte colony-stimulating factor; HIC: high-income country; HLOQ: highest limit of quantification; HP: haptoglobin; IL-10: interleukin 10; IL-18: interleukin 18; IP-10: interferon gamma-induced protein 10; LLOQ: lowest limit of quantification; LTF: lactotransferrin; MA: malarial group; MMP9: matrix metallopeptidase 9; mRDT: malaria rapid diagnostic test; MUAC: middle upper arm circumference; MxA: mixovirus resistance protein A; NGAL: lipocalin; NPS: nasopharyngeal swab; PCA: principal component analysis; PCR: polymerase chain reaction; PCT: procalcitonin; POC: point-of-care; RSV: respiratory syncytial virus; SVM: support vector machine; TIMP-1: tissue inhibitor of metalloproteinases 1; TNFR-2: tumor necrosis factor receptor 2; TRAIL: tumor necrosis factor-related apoptosis-inducing ligand; VI: viral group; WHO: World Health Organization.

For peer review only

Introduction

Clinical pneumonia may be caused by viral or bacterial infections[1] and, where malaria is endemic, malaria can cause a syndrome indistinguishable from clinical pneumonia[2]. There has been a growing number of publications on biomarkers for the diagnosis of bacterial infections and to prognosticate outcomes in patients with clinical pneumonia[3-37]. Such increased attention is driven by the need for low cost point-of-care (POC) tests able to accurately, reliably, and promptly diagnose bacterial infections that can guide treatment [38-41]. All existing tests have delayed turn-around times or require specialized facilities that are often unavailable in resource-limited settings. POC tests can support timely decisions about treatment and ameliorate the over-prescription of antibiotics to treat viral infections, thus slowing the progression of global antibiotic resistance[42-46]. In malaria endemic areas, antibiotics may also be “under-prescribed” and children with bacterial pneumonia may be mistakenly treated for malaria and not receive antibiotic therapy[43 47]. Prognostic POCs could also guide referrals and allow prioritization of intensive treatment measures that are particularly scarce in resource-limited settings.

Symptoms of bacterial and viral diseases and of malaria often overlap[48 49]. Radiographic evidence of pneumonia has been used as an endpoint in studies of pneumococcal vaccine, but chest X-rays, particularly in resource-limited settings, when available have moderate reliability [50-58], and may result in both false positive and negative results. Laboratory tests have low accuracy. Blood or pleural fluid cultures are highly specific for diagnosing bacterial infections but have low sensitivity and results are not promptly available[59 60]. Bacterial polymerase chain reaction (PCR) tests require specialized resources, and have low sensitivity. Viral PCR also requires specialized facilities and do not establish a diagnosis of viral pneumonia[49 61-63]. Furthermore, in children, antigen detection in the urine has limited utility and induced sputum

For peer review only - http://bmjopen.bmj.com/site/about/guidelines.xhtml

samples are difficult to obtain and likely to be contaminated with the normal flora[64-67]. Where malaria is endemic, standard malaria diagnostic tests can detect the parasite in blood. Nevertheless, malaria may co-exist with other infections while not causing the respiratory syndrome[68 69].

Generally, previous biomarker studies to diagnose bacterial diseases or to prognosticate disease in patients with pneumonia or sepsis focused on procalcitonin (PCT) and/or C-reactive protein (CRP) and concluded that the markers had moderate accuracy (area under the receiver operating characteristic curve [AUC-ROC]< 0.80)[9-14 34-36 70-93]. Accuracy of a single biomarker in bacterial pneumonia is probably low, since many biomarkers may be in the inflammatory pathway triggered by several pathogens[94]. More recently, studies screened through novel biomarkers and identified gene transcription signatures able to distinguish patients with bacterial and viral infections[95 96]. Additionally, a biomarker signature based on haptoglobin (HP) and lipocalin (NGAL) has been identified to differentiate malaria and probable bacteria etiology in pediatric patients with clinical pneumonia in a resource-limited country[97]. Two other promising signatures to differentiate viral from bacterial infections in high-income countries (HIC) have been identified. The first (ImmunoXpert) is based on CRP, Tumor Necrosis Factor-related apoptosis-inducing ligand (TRAIL) and Interferon Gamma-Induced Protein 10 (IP-10) and has been validated in HICs, with sensitivity ranging from 87% to 94% and specificity from 90% to 94%[5 98-101].The second is based on CRP and Mixovirus Resistance Protein A (MxA) and has been implemented in a POC (FebriDx) in HICs, with sensitivity and specificity varying from 80% to 96% and 67% to 94%, respectively. [4 102 103] Although promising, these two signatures need to be validated in resource-limited settings where inflammatory responses may vary because of malaria, co-infections, and undernutrition[104].

For peer review only - http://bmjopen.bmj.com/site/about/guidelines.xhtml

In Mozambique, we scanned through several biomarkers and combined them through machine learning methods aiming to detect bacterial infection (versus no bacterial infection) in pediatric patients with clinical pneumonia. Considering the potential of co-existing malaria infections, we sought biomarker signatures by comparing children with malaria, bacterial and viral infections and validated accurate signatures by comparing patients with and without bacterial infections[105]. Signatures with 3-5 proteins were identified that had high sensitivity for the diagnosis of bacterial pneumonia (96-91%), while misdiagnosing few viral cases. The selected signatures performed better than signatures including commonly collected clinical and laboratory markers, as well as pneumonia with consolidation on X-ray. The current study builds on our previous findings and aims to validate previous diagnostic signatures identified by us and other groups, and in addition to improve upon signatures by scanning through novel biomarkers in a different population in rural Gambia.

Biomarkers for signatures will be selected and combined using a comprehensive set of data mining approaches. To validate and improve upon prior signatures and seek prognostic signatures we will add some novel promising biomarkers such as resistin and lactoferrin[37 106 107]. At the end of this study, we expect to select signatures with excellent accuracy (≥ 93% sensitivity), that have the highest prognostic utility, and be comprised of a limited number of biomarkers that can be developed in a POC test. Our focus is on identifying patients who need antibiotic therapy for bacterial diseases.

Study objectives

Specifically, this study aims to:

I. *Assess combinations of blood proteins to accurately and reliably diagnose bacterial pneumonia in pediatric patients.*

For peer review only - http://bmjopen.bmj.com/site/about/guidelines.xhtml

We expect that protein combinations that were identified in previous studies as accurate for diagnosing bacterial infection in patients with clinical pneumonia will have high sensitivity, specificity, and AUC-ROC in an African pediatric population different from the Mozambican children. Biomarkers of these signatures will be absent in healthy children. Moreover, we hypothesize that accuracy of previous signatures can be improved by seeking to combine them with novel candidate proteins.

II. *Explore prognostic biomarker signatures in patients with clinical pneumonia caused by any pathogen.*

We anticipate that combination of inflammatory proteins in the blood measured at admission will accurately identify patients who will clinically deteriorate.

Methods and Analysis

Design

To test existing and identify novel diagnostic biomarker signatures for bacterial disease, we will measure proteins in the blood at admission and collect clinical and laboratory information at admission to ascertain diagnostic groups. Diagnostic signatures will be sought by comparing children in the target diagnosis groups. To seek prognostic biomarker signatures, we will prospectively follow clinical outcomes collected after admission and discharge. When studying prognostic signatures in the primary analysis, proteins will be compared across three groups with poor, moderate and good prognosis. The study started in February of 2019 and is expected to be concluded by November of 2021.

Study Environment

The study will be conducted at the Boston University School of Public Health (BUSPH), the Medical Research Council Unit The Gambia (MRCG) at London School of Hygiene & Tropical

For peer review only - http://bmjopen.bmj.com/site/about/guidelines.xhtml

Medicine, and the Multiplex Core Facility of the Center of Translational Immunology from the University Medical Center Utrecht. Patients will be recruited at Basse and Bansang Hospitals. These hospitals serve a population of 178,510 (224 villages) and 99,113 (217 villages), respectively, with 19% of the population aged <5 years. Diagnostic laboratory tests will be conducted at the MRCG, Basse Field Station. The multiplex bead-based immunoassay will be conducted at CTI/UMC and analysis will be conducted at BUSPH.

For peer review only

Outcomes

Two sets of outcomes will be evaluated, one for each study aim:

Objective I - Diagnostic Group Classification

The primary objective of the diagnostic group classification is to identify patients in need of antibiotic therapy for a bacterial infection and not to identify the pathogen responsible for the respiratory symptoms. Patients will be assigned to bacterial, malarial and viral groups according to a definitive classification (primary analysis) (Fig. 1) when seeking signatures, and bacterial and non-bacterial infection groups when testing signatures. Patients will also be categorized into bacterial and non-bacterial infection groups based on a probable classification criterion (secondary analysis). Patients with ambiguous diagnosis who do not meet our criteria will be excluded from the study of Objective 1. By incorporating the two outcomes, we will assess the validity of the identified biomarker signatures in patients with and without detected bacteremia and provide data to support the generalizability of the inflammatory biomarker signature.

For the definitive classification, a patient will be assigned to the bacterial (BA) group if he/she has a positive bacterial pathogen culture of fluid from a normally sterile site (e.g. blood or pleural fluid). Patients will be assigned to the viral (VI) group if they have negative bacteria microbiological tests, negative malaria blood slides, X-ray without “endpoint pneumonia” (consolidation or pleural effusion[51]), no evidence of fungal infection, and positive PCR for a viral

For peer review only - http://bmjopen.bmj.com/site/about/guidelines.xhtml

pathogen from nasopharyngeal swabs (NPS). Patients will be assigned to the malarial (MA) group if they have normal X-ray (with neither infiltrates nor endpoint pneumonia), no bacterial infection and > 0/μl asexual *P. falciparum* parasites if they are aged < 1 year, or > 2,500 asexual parasites/μl of blood if they are aged > 1 year. Patients who are admitted with viral infections but who develop a secondary bacterial pneumonia during hospitalization will be excluded from the VI group.

Patients with HIV infection or exposure (< 1 year old) will be categorized as fungal pneumonia if they have chest X-ray with bilateral/symmetrical reticular or granular opacities without lobar consolidation and severe hypoxia (≤ 85% at admission) or positive NPS PCR for *Pneumocystis jiroveci* or a blood culture positive to any pathogenic fungal microorganism.

For the diagnostic categorization of probable bacterial pneumonia, patients will be assigned to the probable BA group based on review of data by an expert panel including pediatricians and infectious diseases specialists with at least 10 years of experience. Data will include clinical information collected at and during admission, Chest X-ray results, full blood cell counts, malaria microscopy and/or rapid diagnostic test (mRDT), bacteria culture, viral NPS, and other laboratory test results, when available, e.g., urinalysis. Two experts will review patient data, independently and blindly, classifying patients as “certainly” BA or non-bacteria (NBA), “probable” BA or NBA, “unclear” BA or NBA. Only patients with consensual “certainly” or “probable” diagnosis will be included in this analysis.

Objective II - Poor Prognosis

Poor prognosis will be primarily based on a three-category outcome recorded during and after hospitalization. Additionally, we will explore secondary outcomes associated with a poor

For peer review only - http://bmjopen.bmj.com/site/about/guidelines.xhtml

evolution of the clinical pneumonia during the first 5 days of hospitalization, at discharge and 30 days after admission.

For the three-category outcome, regardless of the original infection, patients will be selected from the following three groups: a) children who die during or up to 30 days after admission; b) children with prolonged hospital stay (> 7 days) or who need to have antibiotic therapy changed within 48 hours of admission, or who are re-admitted within 30 days after the first admission with symptoms associated with the first admission; and c) children discharged well within 3 days of admission and without the need for a change in antibiotic therapy after admission. Secondary outcomes of clinical deterioration will be: a) area under the first five days of the oxygen saturation curve; b) necessity to change antibiotic therapy within the first 2 days from admission or to receive oxygen therapy when it was not required at admission; c) duration of hospital admission.

Participants

We will enroll 900 patients with clinical pneumonia aged 2-59 months by January of 2021 assuming we will have approximately 30 patients with a definitive diagnosis of bacterial infection, 30 with malaria, and 30 with viral infections (Fig. 1). Among the 900 patients, we anticipate enrolling 60 and 100 patients with and without probable bacterial infection, respectively.

For the study of prognostic biomarkers (Fig. 2), we will include 160 patients. All deaths during or up to 30 days from admission will be selected and the remaining sample size will be divided between the other two category outcomes, i.e., (b) prolonged admission, readmission or changed antibiotics during admission, and (c) discharged within 3 days with improvement of

For peer review only - http://bmjopen.bmj.com/site/about/guidelines.xhtml

symptoms. To meet the target sample size in the groups (b) and (c), eligible children will be randomly selected.

A total of 20 community healthy control children aged 2-59 months without any symptoms or signs and no malaria infection will be enrolled in vaccination clinics in Basse to assess precision of signatures.

Patient and Public Involvement

Patients and public were not involved in the design of this research.

Inclusion and Exclusion criteria

Pediatric patients aged 2-59 months will be screened and will be enrolled if they present with cough or difficult breathing, the guardians provide informed consent, are referred for probable admission, and have at least one of the following signs: increased respiratory rate for age based on the World Health Organization (WHO) criteria, lower chest indrawing, peripheral arterial oxygen saturation < 93% (measured by pulse oximetry), grunting, nasal flaring, or undernutrition. The WHO criteria for increased respiratory rate are: respiratory rate ≥ 50 per minute for children 2-11 months old; and respiratory rate ≥ 40 per minute for children 1-5 years old[108]. Undernutrition will be defined as: visible wasting OR middle upper arm circumference (MUAC) < 11.5 cm if aged ≥ 6 months OR weight-for-height Z-scores < -3. Patients will be excluded if they have suspected tuberculosis based on a cough lasting > 2 weeks, were admitted to a hospital in the previous two weeks, and have evidence of any condition that could be worsened by collection of blood.

For peer review only - http://bmjopen.bmj.com/site/about/guidelines.xhtml

Healthy community controls will be enrolled if: a mRDT is negative, vaccines have not been administered that day, there are no symptoms of any disease, and no history of hospital admission in the previous 4 weeks. Healthy controls will be excluded if they are later found to have malaria infection detected by a positive microscopy.

Recruitment and follow-up

Upon arrival at any of the study hospitals, patients aged 2-59 months will be screened for the presence of respiratory symptoms and undernutrition by a Senior Research Nurse with extensive experience in research in pediatric pneumonia (Fig 1). If they pass this first level assessment, they will be referred to a second Research Nurse for a detailed medical history and physical examination. Those patients that are considered eligible will undergo informed consent procedures.

After informed consent, patients will be referred for clinical examination and admission procedures by a Research Clinician and then for sample collection and chest X-ray. NPS will be performed and 5 mL of venous blood will be drawn (never more than 3 mL/kg per WHO standard[109]) with 3 mL immediately placed into one pediatric blood culture bottle (Pedibact®; BD, Franklin Lakes, NJ, USA), and the remaining 2 mL placed in an EDTA vacutainer. From the EDTA tube, a drop of blood will be used for hemoglobin rapid test and mRDT, both part of standard of care in the Gambia. Other tests based on standard of care may be conducted, including urinalyses, glucose, examination of cerebrospinal fluid (CSF) and pleural fluid, or lung aspirate. We will follow local clinical practice and HIV testing will not be routinely conducted. HIV prevalence is approximately 1% in antenatal surveys in The Gambia. Blood in EDTA will be processed and used within one hour from collection for full blood cell count, malaria microscopy, and will be centrifuged and aliquoted to store plasma at -80C in the MRCG biobank for the multiplex bead-based immunoassay. An aliquot of 150µl will be sent to the Center of

For peer review only - http://bmjopen.bmj.com/site/about/guidelines.xhtml

Translational Immunology in Utrecht for quantification of proteins with temperature controlled dry ice shipment.

Follow-up

After admission, patients will be followed daily for 5 days to record: pulse oximetry, antibiotic therapy, feeding, clinical deterioration with need for additional tests. If patients are receiving oxygen during the follow-up visits, they will have their lowest recorded pulse oximetry imputed. At hospital separation, a Research Nurse will fill a case report form (CRF) to record diagnosis and outcome (e.g. death, absconding or discharge alive and well). Moreover, for patients who are discharged, a phone call will be placed to the parent/guardian 30 days after admission.

Study measurements and procedures

- Bacterial Culture: Approximately 3 mL will be incubated in an automatic BACTEC® 9050 system (BD) for a minimum of 4 days. Positive blood cultures will be examined by Gram stain and sub-cultured onto blood agar, chocolate agar or MacConkey agar plates based on Gram stain. Bacterial growth will be defined as contaminant when bacteria generally considered skin flora are isolated from the positive blood culture (e.g., Coagulase–negative Staphylococci, Bacillus species or Micrococci) [110]. Lung fluids will be cultured according to procedures previously described[111].

- Culture of CSF, lung aspirate and pleural fluid aspirate: Cell count and direct gram reaction will be performed as well as bacterial antigen latex agglutination test. A 50 to 100µl aliquot of the specimen will be inoculated onto Blood Agar, Chocolate Agar and MacConkey Agar plates for isolation of bacterial growth.

.

For peer review only - http://bmjopen.bmj.com/site/about/guidelines.xhtml

- Viral PCR of NPS: NPS will be placed in media that will be aliquoted and stored at -80˚C for rtPCR. Assays will use primers and probes for influenza viruses types A and B, respiratory syncytial virus (RSV) types A and B, adenovirus, parainfluenza viruses 1, 2, 3 and 4, human metapneumovirus, coronavirus, bocavirus, swine flu virus (H1N1) and rhinovirus. *P. jiroveci* will also be screened in this PCR. Viral RNA and DNA will be extracted from supernatant of NPS samples using the QIAamp® cador® Pathogen Mini Kit or the Qiacube® HT manufactured by Qiagen and PCR reactions will be ran as described elsewhere[112]. PCR for SARS-CoV-2 is not planned because pediatric pneumonia caused by SARS-CoV-2 is generally rare and clinical cases of COVID-19 in adults have been rare in the region. However, if future evidence suggests that SARS-CoV-2 shall be considered as a pathogen of pediatric clinical pneumonia, SARS-CoV-2 PCR may be performed.

- Malarial Microscopy: Thick blood smear slides will be read blindly by two microscopists and, if discrepant, by a third.

- Full blood cell count (FBC): FBC will be performed on a Medonic M51 5-part hematology analyzer (Boule Diagnostics AB, Domnarvsgatan, Spanga, Sweden). The parameters measured will be white blood cell count, absolute and percent count of lymphocytes, monocytes, neutrophils, eosinophils, basophils, red blood cell count, platelet count, hemoglobin, mean cell volume, hematocrit, red cell distribution width, mean cell hemoglobin, mean corpuscular hemoglobin concentration, and mean platelet volume.

For peer review only - http://bmjopen.bmj.com/site/about/guidelines.xhtml

- Multiplex Bead-Based Immunoassay: Using Luminex® technology will quantify proteins in plasma and will be conducted at the Multiplex Core Facility of the Center of Translational Immunology ISO9001:2015 laboratory using FlexMAP3D systems[113]. A total of 50 proteins will be included in the assay: a) proteins identified in our previous study to constitute accurate signatures (e.g., HP, interleukin 10 and 18 [IL-10/IL-18] and tissue inhibitor of metalloproteinases 1 [TIMP-1], tumor necrosis factor receptor 2 [TNFR-2], and Resistin)[114]; b) proteins that discriminated the three groups in high-throughput scans conducted previously (e.g., bactericidal/permeability-increasing protein [BPI], lactotransferrin [LTF], granulocyte colony-stimulating factor (G-CSF), matrix metallopeptidase 9 [MMP9]); c) proteins specified in previous studies and listed above (e.g., NGAL, PCT, TRAIL, and IP-10); proteins identified in previous studies listed above with prognostic value (e.g., angiopoietin)

- Chest Radiographs: Frontal radiographs will be obtained, and images will be recorded in a computer file. Radiographic findings will be classified as proposed by Cherian et al[51] into presence of consolidation, pleural fluid, other infiltrates, or normal. Additionally, the presence of reticular/reticulonodular patterns, cavities, pneumatoceles, lymphadenopathy, abscess, focal fibrosis, pneumothorax, cardiomegaly, and foreign body will be noted. Chest X-rays will be read by two clinicians and, if discrepant for consolidation, they will be adjudicated by a third reader, all with training in radiographical interpretation for pneumonia and calibration against WHO standard images. Also a pulmonologist, will read a sample of 10% of X-rays for quality assurance and control.

Data management

All data will be captured through laptops/desktop computers using an Electronic Medical Record System (EMRS) developed at the MRCG. The only exception will be for follow-up of admitted

For peer review only - http://bmjopen.bmj.com/site/about/guidelines.xhtml

patients that will be captured on paper forms and later entered in the EMRS. During power outages, paper forms will be completed and when electricity is back, data will be entered into the EMRS. To ensure data quality, data validation rules have been established in the EMRS that generates alert messages. Additionally, a complete process flow has been established to sequentially activate forms for entry and a report listing missing variables is generated during entry of forms to assure form completeness.

Data analysis

Descriptive analysis will be conducted to seek outliers, assess assumptions for tests, and appropriate transformations. Patient severity scores at baseline will be derived based on principal component analysis (PCA), including baseline clinical and laboratory variables. These scores will be included in the analysis and used to explore stratification of patients in the study of diagnostic biomarker signatures. Adjustments for potential confounders (e.g. overall and cell-specific leukocyte counts; breast feeding; undernutrition) and assessment of heterogeneity across subgroups will be done by incorporating the factor in analysis.

In Analysis of Objective 1, we will first test previous diagnostic biomarker signatures[5 105] through estimating AUC-ROC, sensitivity, and specificity with 95% CI. Using levels of biomarkers included in Valim et al[105] and Oved et al[98], we will reproduce signatures using the original models. To discover alternative biomarker signatures, we will start by conducting single biomarker analysis in which we will rank individual markers by pairwise comparisons of the three definitive diagnosis groups (primary analysis) and the two bacterial vs. non-bacterial probable diagnosis groups (secondary analysis) and estimation of AUC-ROC with 95% confidence interval (CI)[115]. We will also estimate sensitivity and specificity for an optimal cutoff (weighting towards high sensitivity to bacteria) based on comparison of the BA and NBA definitive diagnosis groups through resampling. Next, to identify composite biomarkers, all

For peer review only - http://bmjopen.bmj.com/site/about/guidelines.xhtml

For peer review only

markers (including clinical and laboratory tests and severity scores) will be combined and selected through comparison of the three definitive diagnosis groups using: logistic regression with elastic net; classification or regression trees depending on the specific outcome; and support vector machine (SVM). In SVM, non-linear radial basis function kernels will be used and features will be selected through a penalization and recursive feature elimination[116 117]. We will train models and identify signatures based on the three diagnosis groups although the focus is in classification of BA and NBA (defined as MA or VI diagnosis) because using three groups, signatures are likely to be more accurate when markers are not monotonically increasing or decreasing across groups. For instance, if a marker is high in BA, low in VI and high in MA, levels of VI with MA are sub-optimally averaged when VI and MA are collapsed for training signatures. Within each class of models, tuning parameters will be obtained through a metric of misclassification (e.g., deviance or error) estimated in 5-fold cross validation. Additionally, models will be selected based on a biased choice of tuning parameters (those determining ≤ 5 markers). Penalties for missing a bacterial infection will be used. Models will be tested by estimating accuracy to diagnose BA vs. non-BA in the definitive diagnosis classification using resampling. Moreover, accuracy will be estimated by comparing BA with NBA probable diagnosis groups.

Analyses of Objective 2 will use categorical or continuous surrogates of poor prognosis as outcomes and markers as predictors. Markers will be measured at admission. Signatures will be sought and tested using the same models of Objective 1.

To estimate precision, we will report the coefficient of variation (CV) of selected biomarkers in healthy controls. Contaminated cultures will be considered negative. In immunoassays, samples < LLOQ or >HLOQ will be assigned a value below and above the limits of quantification, respectively, through a uniform distribution.

For peer review only - http://bmjopen.bmj.com/site/about/guidelines.xhtml

An α-significance level of 0.05 will be used. In single-marker analysis, p-values and CIs will be adjusted for family-wise error rate (FWER) through permutation or false discovery rate (FDR) through Benjamin-Rocheberg, depending on the analysis[118].

Sample Size Justification

We plan to enroll 900 patients to obtain 30 patients with confirmed bacterial disease based on expectations that ~3% of patients will have a positive bacterial culture for Objective 1. Also, we anticipate that 30 patients will meet definitive viral or malarial etiology. If necessary, we will randomly sample patients from the viral and malarial groups. The choice of 30 patients with and 60 patients without bacterial disease was based on feasibility considerations, as was the choice of 20 healthy controls and 160 patients in the analysis of the "probable" diagnosis categories (a random stratified sample from all available subjects). However, with ~90 patients and only 23 patients with bacterial infections, we could previously identify accurate signatures[105]. We anticipate that in Objective 1, accuracy of previously identified signatures will be estimated with relatively narrow CIs (Table 1). For healthy controls, studying 20 patients will yield a 0.18 CI range for a CV of 0.20 with 0.80 assurance[119]. Analysis of Objective 2 will include a larger number of patients than Objective 1 and shall yield precise confidence intervals and more stable cross-validation based estimates.

For peer review only - http://bmjopen.bmj.com/site/about/guidelines.xhtml

Ethics and Dissemination

Ethics approval has been obtained from the Gambia Government/MRCG Joint Ethics Committee (protocol 1616), and the Institutional Review Board of Boston University Medical Center (STUDY00000958). We plan to share the findings in peer-reviewed journals.

Protocol registration

The study is registered in clinicaltrials.gov (Protocol number H-38462).

Author contributions

The study concept and design were conceived by CV, GM, and PH with contributions of EFK, WDJ, AAD, RCW, JAR, MAM, UDA, QB. Acquisition of clinical data was defined by CV, GM, PH, YAO, YSI, RS, SG, SK and AJ. Acquisiton of laboratory data was defined by CV, RS, EFK, WDJ, RCW, MAM, GM. The analytical plan was conceived by CV. Participant enrollment and follow-up will be conducted by YAO and YSI under direct supervision of GM and oversight from PH, SK, AJ, QB. Sample processing and diagnostic laboratory assays will be supervised by RS, and data management by SG. All on the ground operations in the Gambia will be directly supervised by GM and overseen by CV. QB will be one of the clinicians assigning participants to one of the probable diagnostic groups. The multiplex immunoassay to quantify proteins will be conducted by EK with inputs from CV, WDJ, RCW, JAR, MAM. Analysis will be performed by CV. CV prepared the first draft of the manuscript with input from GM, PH, YAO, YSI, RS, SG. CV, YAO, YAI, RS, SG, EFK, SK, AJ, QB, WDJ, AAD, RCW, JAR, MAM, UDA, PH, and GM provided edits, reviewed, and approved the final version of the manuscript providing substantial intellectual contributions.

Funding

For peer review only

For peer review only - http://bmjopen.bmj.com/site/about/guidelines.xhtml

This study will be supported by the National Institutes of Health (NIH) of the United States (R21AI140258) and will leverage resources from the project entitled “Will the ongoing use of a two-dose, rather than three-dose schedule of pneumococcal conjugate vaccine, have similar impact in rural Gambia” study funded by the Joint Global Health Trials scheme (MRC UK, Welcome, UK AID, UK National Institute for Health Research) and the Bill and Melinda Gates Foundation.

Competing Interests

None declared

Acknowledgement

We thank the contributions of the clinical, laboratory, and data management staff of MRCG to the final version of this study protocol. Furthermore, we are grateful to Dr. Terrie Taylor for her invaluable support, edits, and guidance to this work.

Data Sharing

Data from this study will be available to study investigators and also to the scientific community under request.

For peer review only

Figure 1 – Definitive and probable diagnostic classification criteria (gold standard of diagnostic signature aim) to assign patients to the malaria (MA), bacterial, (BA) and viral (VI) groups (definitive composite criteria) and to the BA and non-bacterial (NBA) groups.

Figure 2 – Exploratory primary (A) and secondary (B) prognosis outcomes.

For peer review only

For peer review only - http://bmjopen.bmj.com/site/about/guidelines.xhtml

Table 1. Precision (exact 95% confidence intervals [CI]) of sensitivity (sample size of 30) and specificity (sample size of 60) when evaluating diagnostic biomarker signatures in analysis of Aim 1.

**Proportion**	**95% CI**	
	**Sensitivity**	**Specificity**
85%	65-94%	73-95%
90%	73-98%	79-90%
95%	78-99%	86-99%

For peer review only

REFERENCES

1. Nair H, Simoes EA, Rudan I, et al. Global and regional burden of hospital admissions for severe acute lower respiratory infections in young children in 2010: a systematic analysis. *Lancet* 2013;381(9875):1380-90. doi: 10.1016/S0140-6736(12)61901-1
2. Taylor WRJ, Hanson J, Turner GDH, et al. Respiratory manifestations of malaria. *Chest* 2012;142(2):492-505. doi: 10.1378/chest.11-2655 [published Online First: 2012/08/09]
3. Tsao TC, Tsai HC, Chang SC. Clinical Usefulness of Urinary Fatty Acid Binding Proteins in Assessing the Severity and Predicting Treatment Response of Pneumonia in Critically Ill Patients: A Cross-Sectional Study. *Medicine* 2016;95(19):e3682. doi: 10.1097/MD.0000000000003682
4. Engelmann I, Dubos F, Lobert PE, et al. Diagnosis of viral infections using myxovirus resistance protein A (MxA). *Pediatrics* 2015;135(4):e985-93. doi: 10.1542/peds.2014-1946
5. van Houten CB, de Groot JAH, Klein A, et al. A host-protein based assay to differentiate between bacterial and viral infections in preschool children (OPPORTUNITY): a double-blind, multicentre, validation study. *Lancet Infect Dis* 2017;17(4):431-40. doi: 10.1016/S1473-3099(16)30519-9
6. Spoorenberg SM, Vestjens SM, Rijkers GT, et al. YKL-40, CCL18 and SP-D predict mortality in patients hospitalized with community-acquired pneumonia. *Respirology* 2017;22(3):542-50. doi: 10.1111/resp.12924 [published Online First: 2016/10/27]
7. Eden E, Srugo I, Gottlieb T, et al. Diagnostic accuracy of a TRAIL, IP-10 and CRP combination for discriminating bacterial and viral etiologies at the Emergency Department. *The Journal of infection* 2016;73(2):177-80. doi: 10.1016/j.jinf.2016.05.002
8. Relster MM, Holm A, Pedersen C. Plasma cytokines eotaxin, MIP-1alpha, MCP-4, and vascular endothelial growth factor in acute lower respiratory tract infection. *APMIS : acta pathologica, microbiologica, et immunologica Scandinavica* 2017;125(2):148-56. doi: 10.1111/apm.12636
9. Higdon MM, Le T, O'Brien KL, et al. Association of C-Reactive Protein With Bacterial and Respiratory Syncytial Virus-Associated Pneumonia Among Children Aged <5 Years in the PERCH Study. *Clinical infectious diseases : an official publication of the Infectious Diseases Society of America* 2017;64(suppl_3):S378-S86. doi: 10.1093/cid/cix150
10. Yan ST, Sun LC, Jia HB, et al. Procalcitonin levels in bloodstream infections caused by different sources and species of bacteria. *The American journal of emergency medicine* 2017;35(4):579-83. doi: 10.1016/j.ajem.2016.12.017
11. McCluskey SM, Schuetz P, Abers MS, et al. Serial Procalcitonin as a Predictor of Bacteremia and Need for Intensive Care Unit Care in Adults With Pneumonia, Including Those With Highest Severity: A Prospective Cohort Study. *Open Forum Infect Dis* 2017;4(1):ofw238. doi: 10.1093/ofid/ofw238 [published Online First: 2017/05/10]
12. Mahende C, Ngasala B, Lusingu J, et al. Profile of C-reactive protein, white cells and neutrophil populations in febrile children from rural north-eastern Tanzania. *The Pan African medical journal* 2017;26:51. doi: 10.11604/pamj.2017.26.51.10264
13. Li YL, Zhai LC, Ji JH, et al. Detection of combined procalcitonin and c-reactive protein applied in the diagnosis of bacterial infections. *Journal of biological regulators and homeostatic agents* 2017;31(1):177-81.
14. Pilotto A, Dini S, Daragjati J, et al. Combined use of the multidimensional prognostic index (MPI) and procalcitonin serum levels in predicting 1-month mortality risk in older patients hospitalized with community-acquired pneumonia (CAP): a prospective study. *Aging clinical and experimental research* 2017 doi: 10.1007/s40520-017-0759-y
15. Andersen SB, Baunbaek Egelund G, Jensen AV, et al. Failure of CRP decline within three days of hospitalization is associated with poor prognosis of Community-acquired

For peer review only - http://bmjopen.bmj.com/site/about/guidelines.xhtml

Pneumonia. *Infect Dis (Lond)* 2017;49(4):251-60. doi: 10.1080/23744235.2016.1253860 [published Online First: 2016/11/27]
16. Chang PY, Tsao SM, Chang JH, et al. Plasma levels of soluble intercellular adhesion molecule-1 as a biomarker for disease severity of patients with community-acquired pneumonia. *Clin Chim Acta* 2016;463:174-80. doi: 10.1016/j.cca.2016.10.030 [published Online First: 2016/12/17]
17. Banoei MM, Vogel HJ, Weljie AM, et al. Plasma metabolomics for the diagnosis and prognosis of H1N1 influenza pneumonia. *Critical care* 2017;21(1):97. doi: 10.1186/s13054-017-1672-7
18. Frenzen FS, Kutschan U, Meiswinkel N, et al. Admission lactate predicts poor prognosis independently of the CRB/CURB-65 scores in community-acquired pneumonia. *Clinical microbiology and infection : the official publication of the European Society of Clinical Microbiology and Infectious Diseases* 2017 doi: 10.1016/j.cmi.2017.07.007
19. Chkhaidze I, Kapanadze N. Cytokines as the Predictors of Severe Mycoplasma Pneumoniae Pneumonia in Children (Review). *Georgian medical news* 2017(267):89-95.
20. Zar HJ, Andronikou S, Nicol MP. Advances in the diagnosis of pneumonia in children. *Bmj* 2017;358:j2739. doi: 10.1136/bmj.j2739
21. Zhou JM, Ye Q. Utility of Assessing Cytokine Levels for the Differential Diagnosis of Pneumonia in a Pediatric Population. *Pediatric critical care medicine : a journal of the Society of Critical Care Medicine and the World Federation of Pediatric Intensive and Critical Care Societies* 2017;18(4):e162-e66. doi: 10.1097/PCC.0000000000001092
22. Curbelo J, Luquero Bueno S, Galvan-Roman JM, et al. Inflammation biomarkers in blood as mortality predictors in community-acquired pneumonia admitted patients: Importance of comparison with neutrophil count percentage or neutrophil-lymphocyte ratio. *PloS one* 2017;12(3):e0173947. doi: 10.1371/journal.pone.0173947
23. Salluh JIF, Souza-Dantas VC, Povoa P. The current status of biomarkers for the diagnosis of nosocomial pneumonias. *Current opinion in critical care* 2017 doi: 10.1097/MCC.0000000000000442
24. Bolatkale M, Duger M, Ulfer G, et al. A novel biochemical marker for community-acquired pneumonia: Ischemia-modified albumin. *The American journal of emergency medicine* 2017;35(8):1121-25. doi: 10.1016/j.ajem.2017.03.018
25. Esposito S, Di Gangi M, Cardinale F, et al. Sensitivity and Specificity of Soluble Triggering Receptor Expressed on Myeloid Cells-1, Midregional Proatrial Natriuretic Peptide and Midregional Proadrenomedullin for Distinguishing Etiology and to Assess Severity in Community-Acquired Pneumonia. *PLoS One* 2016;11(11):e0163262. doi: 10.1371/journal.pone.0163262 [published Online First: 2016/11/16]
26. Li J, Sun L, Xu F, et al. Screening and Identification of APOC1 as a Novel Potential Biomarker for Differentiate of Mycoplasma pneumoniae in Children. *Frontiers in microbiology* 2016;7:1961. doi: 10.3389/fmicb.2016.01961
27. Saghafian-Hedengren S, Mathew JL, Hagel E, et al. Assessment of Cytokine and Chemokine Signatures as Potential Biomarkers of Childhood Community-acquired Pneumonia Severity: A Nested Cohort Study in India. *The Pediatric infectious disease journal* 2017;36(1):102-08. doi: 10.1097/INF.0000000000001364
28. Vogeli A, Ottiger M, Meier MA, et al. Admission levels of asymmetric and symmetric dimethylarginine predict long-term outcome in patients with community-acquired pneumonia. *Respiratory research* 2017;18(1):25. doi: 10.1186/s12931-017-0502-4
29. Vestjens SMT, Spoorenberg SMC, Rijkers GT, et al. High-sensitivity cardiac troponin T predicts mortality after hospitalization for community-acquired pneumonia. *Respirology* 2017;22(5):1000-06. doi: 10.1111/resp.12996 [published Online First: 2017/02/22]
30. Esposito S, Bianchini S, Gambino M, et al. Measurement of lipocalin-2 and syndecan-4 levels to differentiate bacterial from viral infection in children with community-acquired

For peer review only - http://bmjopen.bmj.com/site/about/guidelines.xhtml

pneumonia. *BMC Pulm Med* 2016;16(1):103. doi: 10.1186/s12890-016-0267-4 [published Online First: 2016/07/22]
31. Loonen AJ, Kesarsing C, Kusters R, et al. High pneumococcal DNA load, procalcitonin and suPAR levels correlate to severe disease development in patients with pneumococcal pneumonia. *European journal of clinical microbiology & infectious diseases : official publication of the European Society of Clinical Microbiology* 2017 doi: 10.1007/s10096-017-2963-2
32. Meier MA, Ottiger M, Vogeli A, et al. Activation of the tryptophan/serotonin pathway is associated with severity and predicts outcomes in pneumonia: results of a long-term cohort study. *Clinical chemistry and laboratory medicine* 2017;55(7):1060-69. doi: 10.1515/cclm-2016-0912
33. Zhang Y, Zhou Y, Li S, et al. The Clinical Characteristics and Predictors of Refractory Mycoplasma pneumoniae Pneumonia in Children. *PloS one* 2016;11(5):e0156465. doi: 10.1371/journal.pone.0156465
34. Levy C, Biscardi S, Dommergues MA, et al. Impact of PCV13 on community-acquired pneumonia by C-reactive protein and procalcitonin levels in children. *Vaccine* 2017 doi: 10.1016/j.vaccine.2017.06.057
35. Kim MW, Lim JY, Oh SH. Mortality prediction using serum biomarkers and various clinical risk scales in community-acquired pneumonia. *Scandinavian journal of clinical and laboratory investigation* 2017:1-7. doi: 10.1080/00365513.2017.1344298
36. Stockmann C, Ampofo K, Killpack J, et al. Procalcitonin Accurately Identifies Hospitalized Children With Low Risk of Bacterial Community-Acquired Pneumonia. *Journal of the Pediatric Infectious Diseases Society* 2017 doi: 10.1093/jpids/piw091
37. Irwin AD, Grant A, Williams R, et al. Predicting Risk of Serious Bacterial Infections in Febrile Children in the Emergency Department. *Pediatrics* 2017;140(2) doi: 10.1542/peds.2016-2853
38. Michelow IC, Olsen K, Lozano J, et al. Epidemiology and clinical characteristics of community-acquired pneumonia in hospitalized children. *Pediatrics* 2004;113(4):701-7.
39. English M, Esamai F, Wasunna A, et al. Assessment of inpatient paediatric care in first referral level hospitals in 13 districts in Kenya. *Lancet* 2004;363(9425):1948-53. doi: 10.1016/S0140-6736(04)16408-8
40. Reyburn H, Mwakasungula E, Chonya S, et al. Clinical assessment and treatment in paediatric wards in the north-east of the United Republic of Tanzania. *Bull World Health Organ* 2008;86(2):132-9.
41. Mackenzie G. The definition and classification of pneumonia. *Pneumonia* 2016;8:14. doi: 10.1186/s41479-016-0012-z
42. Donnelly JP, Baddley JW, Wang HE. Antibiotic utilization for acute respiratory tract infections in u.s. Emergency departments. *Antimicrob Agents Chemother* 2014;58(3):1451-7. doi: 10.1128/AAC.02039-13
43. Johansson EW, Selling KE, Nsona H, et al. Integrated paediatric fever management and antibiotic over-treatment in Malawi health facilities: data mining a national facility census. *Malaria journal* 2016;15(1):396. doi: 10.1186/s12936-016-1439-7
44. Ashu EE, Jarju S, Dione M, et al. Population structure, epidemiology and antibiotic resistance patterns of Streptococcus pneumoniae serotype 5: prior to PCV-13 vaccine introduction in Eastern Gambia. *BMC infectious diseases* 2016;16:33. doi: 10.1186/s12879-016-1370-0
45. Makoka MH, Miller WC, Hoffman IF, et al. Bacterial infections in Lilongwe, Malawi: aetiology and antibiotic resistance. *BMC infectious diseases* 2012;12:67. doi: 10.1186/1471-2334-12-67
46. Feasey NA, Masesa C, Jassi C, et al. Three Epidemics of Invasive Multidrug-Resistant Salmonella Bloodstream Infection in Blantyre, Malawi, 1998-2014. *Clinical infectious*

For peer review only - http://bmjopen.bmj.com/site/about/guidelines.xhtml

diseases : an official publication of the Infectious Diseases Society of America 2015;61 Suppl 4:S363-71. doi: 10.1093/cid/civ691
47. Gwer S, Newton CR, Berkley JA. Over-diagnosis and co-morbidity of severe malaria in African children: a guide for clinicians. *Am J Trop Med Hyg* 2007;77(6 Suppl):6-13.
48. Bassat Q, Machevo S, O'Callaghan-Gordo C, et al. Distinguishing malaria from severe pneumonia among hospitalized children who fulfilled integrated management of childhood illness criteria for both diseases: a hospital-based study in Mozambique. *Am J Trop Med Hyg* 2011;85(4):626-34. doi: 10.4269/ajtmh.2011.11-0223
49. Ramirez M, Melo-Cristino J. Expanding the diagnosis of pediatric bacteremic pneumococcal pneumonia from blood cultures to molecular methods: advantages and caveats. *Clin Infect Dis* 2010;51(9):1050-2. doi: 10.1086/656580
50. Bradley JS, Byington CL, Shah SS, et al. The management of community-acquired pneumonia in infants and children older than 3 months of age: clinical practice guidelines by the Pediatric Infectious Diseases Society and the Infectious Diseases Society of America. *Clin Infect Dis* 2011;53(7):e25-76. doi: 10.1093/cid/cir531
51. Cherian T, Mulholland EK, Carlin JB, et al. Standardized interpretation of paediatric chest radiographs for the diagnosis of pneumonia in epidemiological studies. *Bull World Health Organ* 2005;83(5):353-9. doi: /S0042-96862005000500011
52. Edwards M, Lawson Z, Morris S, et al. The presence of radiological features on chest radiographs: how well do clinicians agree? *Clin Radiol* 2012;67(7):664-8. doi: 10.1016/j.crad.2011.12.003
53. Davies HD, Wang EE, Manson D, et al. Reliability of the chest radiograph in the diagnosis of lower respiratory infections in young children. *Pediatr Infect Dis J* 1996;15(7):600-4.
54. Albaum MN, Hill LC, Murphy M, et al. Interobserver reliability of the chest radiograph in community-acquired pneumonia. PORT Investigators. *Chest* 1996;110(2):343-50.
55. Hagaman JT, Rouan GW, Shipley RT, et al. Admission chest radiograph lacks sensitivity in the diagnosis of community-acquired pneumonia. *Am J Med Sci* 2009;337(4):236-40. doi: 10.1097/MAJ.0b013e31818ad805
56. Johnson J, Kline JA. Intraobserver and interobserver agreement of the interpretation of pediatric chest radiographs. *Emerg Radiol* 2010;17(4):285-90. doi: 10.1007/s10140-009-0854-2
57. Loeb MB, Carusone SB, Marrie TJ, et al. Interobserver reliability of radiologists' interpretations of mobile chest radiographs for nursing home-acquired pneumonia. *J Am Med Dir Assoc* 2006;7(7):416-9. doi: 10.1016/j.jamda.2006.02.004
58. O'Grady KA, Torzillo PJ, Ruben AR, et al. Identification of radiological alveolar pneumonia in children with high rates of hospitalized respiratory infections: comparison of WHO-defined and pediatric pulmonologist diagnosis in the clinical context. *Pediatr Pulmonol* 2012;47(4):386-92. doi: 10.1002/ppul.21551
59. Murdoch DR, O'Brien KL, Scott JA, et al. Breathing new life into pneumonia diagnostics. *J Clin Microbiol* 2009;47(11):3405-8. doi: 10.1128/JCM.01685-09
60. Gonsalves WI, Cornish N, Moore M, et al. Effects of volume and site of blood draw on blood culture results. *J Clin Microbiol* 2009;47(11):3482-5. doi: 10.1128/JCM.02107-08
61. Avni T, Mansur N, Leibovici L, et al. PCR using blood for diagnosis of invasive pneumococcal disease: systematic review and meta-analysis. *J Clin Microbiol* 2010;48(2):489-96. doi: 10.1128/JCM.01636-09
62. Resti M, Moriondo M, Cortimiglia M, et al. Community-acquired bacteremic pneumococcal pneumonia in children: diagnosis and serotyping by real-time polymerase chain reaction using blood samples. *Clin Infect Dis* 2010;51(9):1042-9. doi: 10.1086/656579
63. Morpeth SC, Deloria Knoll M, Scott JAG, et al. Detection of Pneumococcal DNA in Blood by Polymerase Chain Reaction for Diagnosing Pneumococcal Pneumonia in Young Children From Low- and Middle-Income Countries. *Clinical infectious diseases : an*

For peer review only - http://bmjopen.bmj.com/site/about/guidelines.xhtml

official publication of the Infectious Diseases Society of America 2017;64(suppl_3):S347-S56. doi: 10.1093/cid/cix145
64. Anjay MA, Anoop P. Diagnostic utility of rapid immunochromatographic urine antigen testing in suspected pneumococcal infections. *Archives of disease in childhood* 2008;93(7):628-31. doi: 10.1136/adc.2008.138958
65. Charkaluk ML, Kalach N, Mvogo H, et al. Assessment of a rapid urinary antigen detection by an immunochromatographic test for diagnosis of pneumococcal infection in children. *Diagn Microbiol Infect Dis* 2006;55(2):89-94. doi: 10.1016/j.diagmicrobio.2005.10.013
66. Thea DM, Seidenberg P, Park DE, et al. Limited Utility of Polymerase Chain Reaction in Induced Sputum Specimens for Determining the Causes of Childhood Pneumonia in Resource-Poor Settings: Findings From the Pneumonia Etiology Research for Child Health (PERCH) Study. *Clinical infectious diseases : an official publication of the Infectious Diseases Society of America* 2017;64(suppl_3):S289-S300. doi: 10.1093/cid/cix098
67. Murdoch DR, Morpeth SC, Hammitt LL, et al. The Diagnostic Utility of Induced Sputum Microscopy and Culture in Childhood Pneumonia. *Clinical infectious diseases : an official publication of the Infectious Diseases Society of America* 2017;64(suppl_3):S280-S88. doi: 10.1093/cid/cix090
68. Koram KA, Molyneux ME. When is "malaria" malaria? The different burdens of malaria infecttion, malaria disease, and malaria-like illnesses. . *Am J Trop Med Hyg* 2007;77(Suppl 6):1-5.
69. D'Acremont V, Kilowoko M, Kyungu E, et al. Beyond malaria--causes of fever in outpatient Tanzanian children. *N Engl J Med* 2014;370(9):809-17. doi: 10.1056/NEJMoa1214482
70. Maniaci V, Dauber A, Weiss S, et al. Procalcitonin in young febrile infants for the detection of serious bacterial infections. *Pediatrics* 2008;122(4):701-10. doi: 10.1542/peds.2007-3503
71. Nascimento-Carvalho CM, Cardoso MR, Barral A, et al. Procalcitonin is useful in identifying bacteraemia among children with pneumonia. *Scandinavian journal of infectious diseases* 2010;42(9):644-9. doi: 10.3109/00365541003796775
72. Zhydkov A, Christ-Crain M, Thomann R, et al. Utility of procalcitonin, C-reactive protein and white blood cells alone and in combination for the prediction of clinical outcomes in community-acquired pneumonia. *Clinical chemistry and laboratory medicine* 2015;53(4):559-66. doi: 10.1515/cclm-2014-0456
73. Tang BM, Eslick GD, Craig JC, et al. Accuracy of procalcitonin for sepsis diagnosis in critically ill patients: systematic review and meta-analysis. *Lancet Infect Dis* 2007;7(3):210-7. doi: 10.1016/S1473-3099(07)70052-X
74. Thayyil S, Shenoy M, Hamaluba M, et al. Is procalcitonin useful in early diagnosis of serious bacterial infections in children? *Acta Paediatr* 2005;94(2):155-8.
75. Kruger S, Ewig S, Marre R, et al. Procalcitonin predicts patients at low risk of death from community-acquired pneumonia across all CRB-65 classes. *Eur Respir J* 2008;31(2):349-55. doi: 10.1183/09031936.00054507
76. Hirakata Y, Yanagihara K, Kurihara S, et al. Comparison of usefulness of plasma procalcitonin and C-reactive protein measurements for estimation of severity in adults with community-acquired pneumonia. *Diagnostic microbiology and infectious disease* 2008;61(2):170-4. doi: 10.1016/j.diagmicrobio.2008.01.014
77. Meili M, Kutz A, Briel M, et al. Infection biomarkers in primary care patients with acute respiratory tract infections-comparison of Procalcitonin and C-reactive protein. *BMC pulmonary medicine* 2016;16:43. doi: 10.1186/s12890-016-0206-4
78. Lindstrom ST, Wong EK. Procalcitonin, a valuable biomarker assisting clinical decision-making in the management of community-acquired pneumonia. *Internal medicine journal* 2014;44(4):390-7. doi: 10.1111/imj.12374

For peer review only - http://bmjopen.bmj.com/site/about/guidelines.xhtml

79. Heiskanen-Kosma T, Korppi M. Serum C-reactive protein cannot differentiate bacterial and viral aetiology of community-acquired pneumonia in children in primary healthcare settings. *Scand J Infect Dis* 2000;32(4):399-402.
80. Korppi M, Remes S, Heiskanen-Kosma T. Serum procalcitonin concentrations in bacterial pneumonia in children: a negative result in primary healthcare settings. *Pediatr Pulmonol* 2003;35(1):56-61. doi: 10.1002/ppul.10201
81. Rodriguez AH, Aviles-Jurado FX, Diaz E, et al. Procalcitonin (PCT) levels for ruling-out bacterial coinfection in ICU patients with influenza: A CHAID decision-tree analysis. *The Journal of infection* 2016;72(2):143-51. doi: 10.1016/j.jinf.2015.11.007
82. Haran JP, Beaudoin FL, Suner S, et al. C-reactive protein as predictor of bacterial infection among patients with an influenza-like illness. *Am J Emerg Med* 2013;31(1):137-44. doi: 10.1016/j.ajem.2012.06.026
83. Johansson N, Kalin M, Backman-Johansson C, et al. Procalcitonin levels in community-acquired pneumonia - correlation with aetiology and severity. *Scand J Infect Dis* 2014;46(11):787-91. doi: 10.3109/00365548.2014.945955
84. Schuetz P, Batschwaroff M, Dusemund F, et al. Effectiveness of a procalcitonin algorithm to guide antibiotic therapy in respiratory tract infections outside of study conditions: a post-study survey. *European journal of clinical microbiology & infectious diseases : official publication of the European Society of Clinical Microbiology* 2010;29(3):269-77. doi: 10.1007/s10096-009-0851-0
85. Long W, Deng X, Zhang Y, et al. Procalcitonin guidance for reduction of antibiotic use in low-risk outpatients with community-acquired pneumonia. *Respirology* 2011;16(5):819-24. doi: 10.1111/j.1440-1843.2011.01978.x
86. Christ-Crain M, Stolz D, Bingisser R, et al. Procalcitonin guidance of antibiotic therapy in community-acquired pneumonia: a randomized trial. *American journal of respiratory and critical care medicine* 2006;174(1):84-93. doi: 10.1164/rccm.200512-1922OC
87. Huang DT, Weissfeld LA, Kellum JA, et al. Risk prediction with procalcitonin and clinical rules in community-acquired pneumonia. *Annals of emergency medicine* 2008;52(1):48-58 e2. doi: 10.1016/j.annemergmed.2008.01.003
88. Don M, Valent F, Korppi M, et al. Efficacy of serum procalcitonin in evaluating severity of community-acquired pneumonia in childhood. *Scand J Infect Dis* 2007;39(2):129-37. doi: 10.1080/00365540600951283
89. Michelow IC, Katz K, McCracken GH, et al. Systemic cytokine profile in children with community-acquired pneumonia. *Pediatric pulmonology* 2007;42(7):640-5. doi: 10.1002/ppul.20633
90. Florin TA, Ambroggio L. Biomarkers for community-acquired pneumonia in the emergency department. *Curr Infect Dis Rep* 2014;16(12):451. doi: 10.1007/s11908-014-0451-8
91. Muller B, Harbarth S, Stolz D, et al. Diagnostic and prognostic accuracy of clinical and laboratory parameters in community-acquired pneumonia. *BMC Infect Dis* 2007;7:10. doi: 10.1186/1471-2334-7-10
92. Bafadhel M, Clark TW, Reid C, et al. Procalcitonin and C-reactive protein in hospitalized adult patients with community-acquired pneumonia or exacerbation of asthma or COPD. *Chest* 2011;139(6):1410-18. doi: 10.1378/chest.10-1747
93. Principi N, Esposito S. Biomarkers in Pediatric Community-Acquired Pneumonia. *International journal of molecular sciences* 2017;18(2) doi: 10.3390/ijms18020447
94. Bordon J, Aliberti S, Fernandez-Botran R, et al. Understanding the roles of cytokines and neutrophil activity and neutrophil apoptosis in the protective versus deleterious inflammatory response in pneumonia. *Int J Infect Dis* 2013;17(2):e76-83. doi: 10.1016/j.ijid.2012.06.006 [published Online First: 2012/10/17]

For peer review only - http://bmjopen.bmj.com/site/about/guidelines.xhtml

95. Mahajan P, Kuppermann N, Mejias A, et al. Association of RNA Biosignatures With Bacterial Infections in Febrile Infants Aged 60 Days or Younger. *JAMA* 2016;316(8):846-57. doi: 10.1001/jama.2016.9207 [published Online First: 2016/08/24]
96. Herberg JA, Kaforou M, Wright VJ, et al. Diagnostic Test Accuracy of a 2-Transcript Host RNA Signature for Discriminating Bacterial vs Viral Infection in Febrile Children. *JAMA* 2016;316(8):835-45. doi: 10.1001/jama.2016.11236 [published Online First: 2016/08/24]
97. Huang H, Ideh RC, Gitau E, et al. Discovery and Validation of Biomarkers to Guide Clinical Management of Pneumonia in African Children. *Clin Infect Dis* 2014 doi: 10.1093/cid/ciu202
98. Oved K, Cohen A, Boico O, et al. A novel host-proteome signature for distinguishing between acute bacterial and viral infections. *PLoS One* 2015;10(3):e0120012. doi: 10.1371/journal.pone.0120012 [published Online First: 2015/03/19]
99. Srugo I, Klein A, Stein M, et al. Validation of a Novel Assay to Distinguish Bacterial and Viral Infections. *Pediatrics* 2017;140(4) doi: 10.1542/peds.2016-3453 [published Online First: 2017/09/15]
100. Stein M, Lipman-Arens S, Oved K, et al. A novel host-protein assay outperforms routine parameters for distinguishing between bacterial and viral lower respiratory tract infections. *Diagn Microbiol Infect Dis* 2018;90(3):206-13. doi: 10.1016/j.diagmicrobio.2017.11.011 [published Online First: 2017/12/24]
101. Ashkenazi-Hoffnung L, Oved K, Navon R, et al. A host-protein signature is superior to other biomarkers for differentiating between bacterial and viral disease in patients with respiratory infection and fever without source: a prospective observational study. *Eur J Clin Microbiol Infect Dis* 2018;37(7):1361-71. doi: 10.1007/s10096-018-3261-3 [published Online First: 2018/04/28]
102. Self WH, Rosen J, Sharp SC, et al. Diagnostic Accuracy of FebriDx: A Rapid Test to Detect Immune Responses to Viral and Bacterial Upper Respiratory Infections. *J Clin Med* 2017;6(10) doi: 10.3390/jcm6100094 [published Online First: 2017/10/11]
103. Shapiro NI, Self WH, Rosen J, et al. A prospective, multi-centre US clinical trial to determine accuracy of FebriDx point-of-care testing for acute upper respiratory infections with and without a confirmed fever. *Ann Med* 2018;50(5):420-29. doi: 10.1080/07853890.2018.1474002 [published Online First: 2018/05/19]
104. Dittrich S, Tadesse BT, Moussy F, et al. Target Product Profile for a Diagnostic Assay to Differentiate between Bacterial and Non-Bacterial Infections and Reduce Antimicrobial Overuse in Resource-Limited Settings: An Expert Consensus. *PLoS One* 2016;11(8):e0161721. doi: 10.1371/journal.pone.0161721 [published Online First: 2016/08/26]
105. Valim C, Ahmad R, Lanaspa M, et al. Responses to Bacteria, Virus, and Malaria Distinguish the Etiology of Pediatric Clinical Pneumonia. *Am J Respir Crit Care Med* 2016;193(4):448-59. doi: 10.1164/rccm.201506-1100OC [published Online First: 2015/10/16]
106. Irwin AD, Marriage F, Mankhambo LA, et al. Novel biomarker combination improves the diagnosis of serious bacterial infections in Malawian children. *BMC Med Genomics* 2012;5:13. doi: 10.1186/1755-8794-5-13 [published Online First: 2012/05/09]
107. Berlutti F, Pantanella F, Natalizi T, et al. Antiviral properties of lactoferrin--a natural immunity molecule. *Molecules* 2011;16(8):6992-7018. doi: 10.3390/molecules16086992 [published Online First: 2011/08/19]
108. WHO. Integrated Management of Childhood Illness. Geneva: World Health Organization 2014.
109. Howie SR. Blood sample volumes in child health research: review of safe limits. *Bull World Health Organ* 2011;89(1):46-53. doi: 10.2471/BLT.10.080010

For peer review only - http://bmjopen.bmj.com/site/about/guidelines.xhtml

110. Bates DW, Lee TH. Rapid classification of positive blood cultures. Prospective validation of a multivariate algorithm. *JAMA* 1992;267(14):1962-6. [published Online First: 1992/04/08]
111. Adegbola RA, Falade AG, Sam BE, et al. The etiology of pneumonia in malnourished and well-nourished Gambian children. *The Pediatric infectious disease journal* 1994;13(11):975-82.
112. Gunson RN, Collins TC, Carman WF. Real-time RT-PCR detection of 12 respiratory viral infections in four triplex reactions. *J Clin Virol* 2005;33(4):341-4. doi: 10.1016/j.jcv.2004.11.025 [published Online First: 2005/06/02]
113. Scholman RC, Giovannone B, Hiddingh S, et al. Effect of anticoagulants on 162 circulating immune related proteins in healthy subjects. *Cytokine* 2018;106:114-24. doi: 10.1016/j.cyto.2017.10.021 [published Online First: 2017/11/02]
114. Valim C, Ahmad R, Lanaspa M, et al. Responses to Bacteria, Virus and Malaria Distinguish the Etiology of Pediatric Clinical Pneumonia. *Am J Respir Crit Care Med* 2015 doi: 10.1164/rccm.201506-1100OC
115. DeLong ER, DeLong DM, Clarke-Pearson DL. Comparing the areas under two or more correlated receiver operating characteristic curves: a nonparametric approach. *Biometrics* 1988;44(3):837-45.
116. Guyon I, Weston J, Barnhill S, et al. Gene Selection for Cancer Classification using Support Vector Machines. *Machine Learning* 2002;46(1-3):389-422. doi: 10.1023/A:1012487302797
117. Becker N, Toedt G, Lichter P, et al. Elastic SCAD as a novel penalization method for SVM classification tasks in high-dimensional data. *BMC Bioinformatics* 2011;12:138. doi: 10.1186/1471-2105-12-138
118. Goeman JJ, Solari A. Multiple hypothesis testing in genomics. *Stat Med* 2014;33(11):1946-78. doi: 10.1002/sim.6082
119. Kelley K. Sample size planning for the coefficient of variation from the accuracy in parameter estimation approach. *Behavior research methods* 2007;39(4):755-66.

For peer review only

For peer review only - http://bmjopen.bmj.com/site/about/guidelines.xhtml

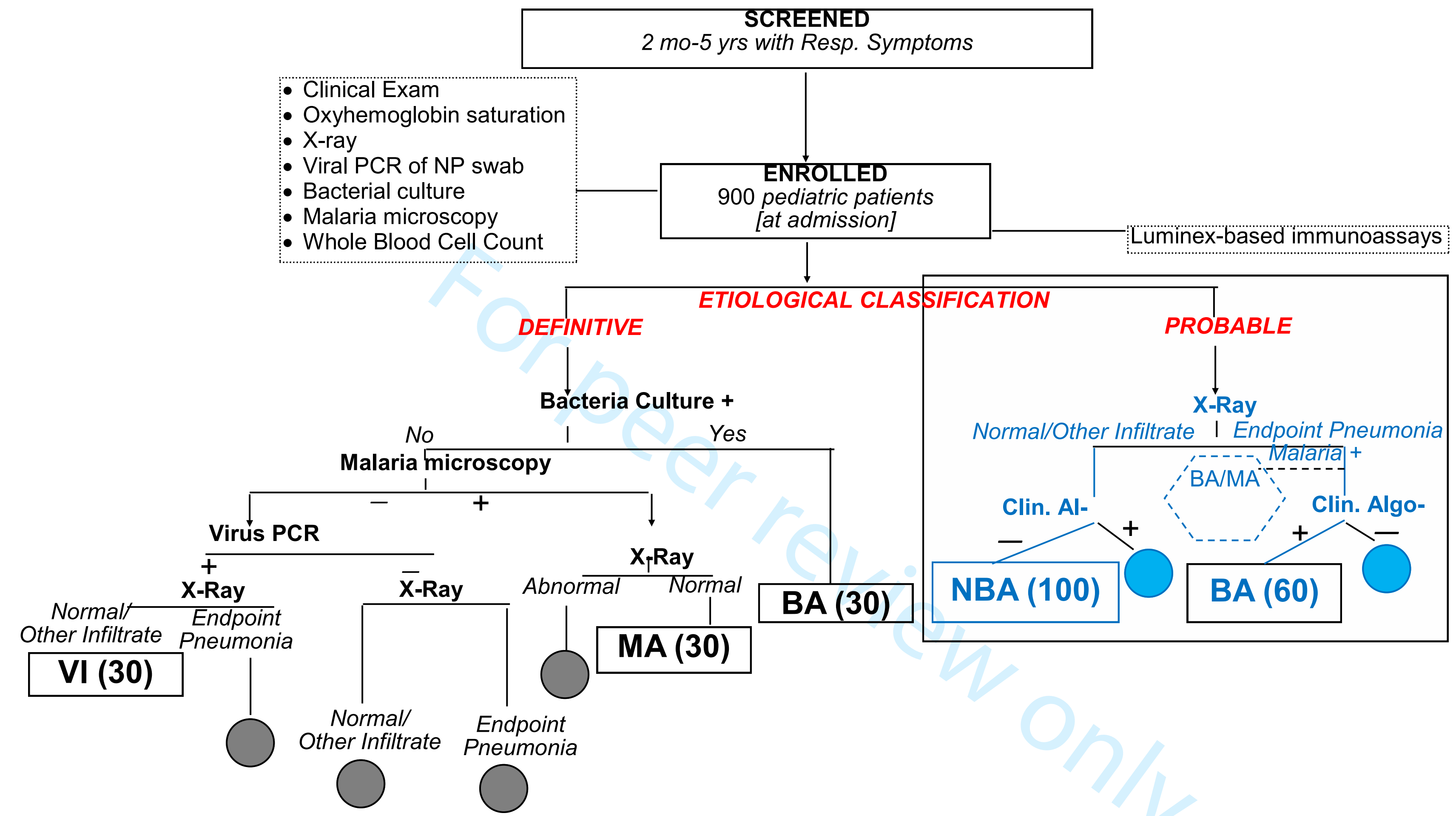

Ambiguos classification groups

Endpoint pneumonia: consolidation or pleural effusion.

For peer review only

3 PROGNOSIS GROUPS (N = 160)		
**Poor prognosis**	**Adverse progress of disease**	**Good Prognosis**
• Dearth	• Re-admission<br>• Need to switch antibiotic therapy ≤ 48 hrs of hospitalization<br>• > 3 days of hospitalization	• Discharged well<br>• No need to switch antibiotic therapy during admission<br>• < 3 days of hospitaliza-

CONTINUOUS AND CATEGORICAL POOR PROGNOSIS OUTCOMES (N = 160)
• Area under 5-day SaO_2<br>• Need to switch antibiotic therapy ≤ 48 hrs of hospitalization<br>• Length of hospital stay

For peer review only

Section & Topic	No	Item
**TITLE OR ABSTRACT**		
	1	Identification as a study of diagnostic accuracy using at least one measure of accuracy (such as sensitivity, specificity, predictive values, or AUC)
**ABSTRACT**		
	2	Structured summary of study design, methods, results, and conclusions (for specific guidance, see STARD for Abstracts)
**INTRODUCTION**		
	3	Scientific and clinical background, including the intended use and clinical role of the index test
	4	Study objectives and hypotheses
**METHODS**		
Study design	5	Whether data collection was planned before the index test and reference standard were performed (prospective study) or after (retrospective study)
Participants	6	Eligibility criteria
	7	On what basis potentially eligible participants were identified (such as symptoms, results from previous tests, inclusion in registry)
	8	Where and when potentially eligible participants were identified (setting, location and dates)
	9	Whether participants formed a consecutive, random or convenience series
Test methods	10a	Index test, in sufficient detail to allow replication
	10b	Reference standard, in sufficient detail to allow replication
	11	Rationale for choosing the reference standard (if alternatives exist)
	12a	Definition of and rationale for test positivity cut-offs or result categories of the index test, distinguishing pre-specified from exploratory
	12b	Definition of and rationale for test positivity cut-offs or result categories of the reference standard, distinguishing pre-specified from exploratory
	13a	Whether clinical information and reference standard results were available to the performers/readers of the index test
	13b	Whether clinical information and index test results were available to the assessors of the reference standard
Analysis	14	Methods for estimating or comparing measures of diagnostic accuracy
	15	How indeterminate index test or reference standard results were handled
	16	How missing data on the index test and reference standard were handled
	17	Any analyses of variability in diagnostic accuracy, distinguishing pre-specified from exploratory
	18	Intended sample size and how it was determined
**RESULTS**		
Participants	19	Flow of participants, using a diagram
	20	Baseline demographic and clinical characteristics of participants
	21a	Distribution of severity of disease in those with the target condition
	21b	Distribution of alternative diagnoses in those without the target condition
	22	Time interval and any clinical interventions between index test and reference standard
Test results	23	Cross tabulation of the index test results (or their distribution) by the results of the reference standard
	24	Estimates of diagnostic accuracy and their precision (such as 95% confidence intervals)
	25	Any adverse events from performing the index test or the reference standard
**DISCUSSION**		
	26	Study limitations, including sources of potential bias, statistical uncertainty, and generalisability
	27	Implications for practice, including the intended use and clinical role of the index test
**OTHER INFORMATION**		
	28	Registration number and name of registry
	29	Where the full study protocol can be accessed
	30	Sources of funding and other support; role of funders

For peer review only - http://bmjopen.bmj.com/site/about/guidelines.xhtml

STARD 2015

AIM

STARD stands for "Standards for Reporting Diagnostic accuracy studies". This list of items was developed to contribute to the completeness and transparency of reporting of diagnostic accuracy studies. Authors can use the list to write informative study reports. Editors and peer-reviewers can use it to evaluate whether the information has been included in manuscripts submitted for publication.

EXPLANATION

A **diagnostic accuracy study** evaluates the ability of one or more medical tests to correctly classify study participants as having a **target condition.** This can be a disease, a disease stage, response or benefit from therapy, or an event or condition in the future. A medical test can be an imaging procedure, a laboratory test, elements from history and physical examination, a combination of these, or any other method for collecting information about the current health status of a patient.

The test whose accuracy is evaluated is called **index test.** A study can evaluate the accuracy of one or more index tests. Evaluating the ability of a medical test to correctly classify patients is typically done by comparing the distribution of the index test results with those of the **reference standard**. The reference standard is the best available method for establishing the presence or absence of the target condition. An accuracy study can rely on one or more reference standards.

If test results are categorized as either positive or negative, the cross tabulation of the index test results against those of the reference standard can be used to estimate the **sensitivity** of the index test (the proportion of participants *with* the target condition who have a positive index test), and its **specificity** (the proportion *without* the target condition who have a negative index test). From this cross tabulation (sometimes referred to as the contingency or "2x2" table), several other accuracy statistics can be estimated, such as the positive and negative **predictive values** of the test. Confidence intervals around estimates of accuracy can then be calculated to quantify the statistical **precision** of the measurements.

If the index test results can take more than two values, categorization of test results as positive or negative requires a **test positivity cut-off**. When multiple such cut-offs can be defined, authors can report a receiver operating characteristic (ROC) curve which graphically represents the combination of sensitivity and specificity for each possible test positivity cut-off. The **area under the ROC curve** informs in a single numerical value about the overall diagnostic accuracy of the index test.

The **intended use** of a medical test can be diagnosis, screening, staging, monitoring, surveillance, prediction or prognosis. The **clinical role** of a test explains its position relative to existing tests in the clinical pathway. A replacement test, for example, replaces an existing test. A triage test is used before an existing test; an add-on test is used after an existing test.

Besides diagnostic accuracy, several other outcomes and statistics may be relevant in the evaluation of medical tests. Medical tests can also be used to classify patients for purposes other than diagnosis, such as staging or prognosis. The STARD list was not explicitly developed for these other outcomes, statistics, and study types, although most STARD items would still apply.

DEVELOPMENT

This STARD list was released in 2015. The 30 items were identified by an international expert group of methodologists, researchers, and editors. The guiding principle in the development of STARD was to select items that, when reported, would help readers to judge the potential for bias in the study, to appraise the applicability of the study findings and the validity of conclusions and recommendations. The list represents an update of the first version, which was published in 2003.

More information can be found on http://www.equator-network.org/reporting-guidelines/stard.

For peer review only
